# Transcriptomic time-series analysis of cold- and heat-shock response in psychrotrophic lactic acid bacteria

**DOI:** 10.1186/s12864-020-07338-8

**Published:** 2021-01-07

**Authors:** Ilhan Cem Duru, Anne Ylinen, Sergei Belanov, Alan Avila Pulido, Lars Paulin, Petri Auvinen

**Affiliations:** Institute of Biotechnology, University of Helsinki, Helsinki, Finland

**Keywords:** RNA-seq, Gene network inference, Time-series, Differential gene expression, Stress, Psychrotrophic lactic acid bacteria, Cold and heat shock

## Abstract

**Background:**

Psychrotrophic lactic acid bacteria (LAB) species are the dominant species in the microbiota of cold-stored modified-atmosphere-packaged food products and are the main cause of food spoilage. Despite the importance of psychrotrophic LAB, their response to cold or heat has not been studied. Here, we studied the transcriptome-level cold- and heat-shock response of spoilage lactic acid bacteria with time-series RNA-seq for *Le. gelidum, Lc. piscium*, and *P. oligofermentans* at 0 °C, 4 °C, 14 °C, 25 °C, and 28 °C.

**Results:**

We observed that the cold-shock protein A (*cspA*) gene was the main cold-shock protein gene in all three species. Our results indicated that DEAD-box RNA helicase genes (*cshA*, *cshB*) also play a critical role in cold-shock response in psychrotrophic LAB. In addition, several RNase genes were involved in cold-shock response in *Lc. piscium* and *P. oligofermentans.* Moreover, gene network inference analysis provided candidate genes involved in cold-shock response. Ribosomal proteins, tRNA modification, rRNA modification, and ABC and efflux MFS transporter genes clustered with cold-shock response genes in all three species, indicating that these genes could be part of the cold-shock response machinery. Heat-shock treatment caused upregulation of Clp protease and chaperone genes in all three species. We identified transcription binding site motifs for heat-shock response genes in *Le. gelidum* and *Lc. piscium.* Finally, we showed that food spoilage-related genes were upregulated at cold temperatures.

**Conclusions:**

The results of this study provide new insights on the cold- and heat-shock response of psychrotrophic LAB. In addition, candidate genes involved in cold- and heat-shock response predicted using gene network inference analysis could be used as targets for future studies.

**Supplementary Information:**

The online version contains supplementary material available at 10.1186/s12864-020-07338-8.

## Background

Lactic acid bacteria (LAB) are a group of gram-positive bacteria with a wide range of phenotypic and genomic features [1]. LAB communities play an important role in fermented foods during the production stage and can be also used as food preservatives [2]. Furthermore, psychrotrophic LAB cause food spoilage in cold-stored modified-atmosphere-packaged (MAP) food products, since they are able to prevail in the MAP food environment [3]. LAB species composition and their relative abundance depend on the nature of the food product and preservation technology [4, 5]. However, two LAB species, *Leuconostoc gelidum* and *Lactococcus piscium*, have been found to frequently predominate at the end of the shelf life in a variety of packaged and refrigerated foods of animal and plant origin [6–9]. Spoilage communities also contain less abundant and slower growing species, such as *Paucilactobacillus oligofermentans* (formerly *Lactobacillus oligofermentans*), the role of which in food spoilage is unclear [10, 11]. We have been investigating these three LAB species for several years and have sequenced their genomes [12–15] and analyzed their gene expression patterns in growth experiments [14–16]. Since reverse genetics methods are not efficient for these species, detailed omics analysis is the best way to study them. Understanding gene expression mechanisms of these spoilage LAB is important, since MAP technology with combined cold storage has increased its popularity for preservation of minimally processed fresh foods. A better understanding of LAB genomics and especially mechanisms of cold-shock and stress adaptation is crucial for discovery of new methods of spoilage control.

There are three main categories of bacteria based on their ability to grow at different temperatures. These are thermophiles, mesophiles, and psychrophiles that are able to grow at high, intermediate, and low temperatures, respectively [17, 18]. Psychrophiles are categorized into psychrophiles sensu stricto, which optimally grow at 15 °C, and psychrotrophic (psychrotolerant), which optimally grow at 20–25 °C [19–21]. Based on previously published studies, cold-shock protein (CSP), DEAD-box RNA helicase, and ribonuclease (RNase) are commonly known cold-shock response gene families in all three types of bacteria [22–25]. Similarly, chaperone and Clp gene families are the common heat-shock response genes in bacteria [18, 26]. To our knowledge, although the cold- and heat-shock response has been previously investigated in mesophilic LAB [27–29], these responses have not been investigated in psychrotrophic LAB. Here, to investigate both cold- and heat-shock response in spoilage psychrotrophic LAB, we performed RNA-seq using five temperatures (0 °C, 4 °C, 14 °C, 25 °C, and 28 °C) and three timepoints (5, 35, 185 min) for each temperature. The timepoints were selected to capture early and also later effects of temperature change, while keeping the sample number reasonable. Temperatures were selected based on literature analysis of the biology of psychrotrophic bacteria [19–21]. Previous studies showed that the optimal temperature for *Le. gelidum* and *Lc. piscium* is 25 °C [6, 30]. The two lowest temperatures used (0 °C and 4 °C) cause cold-shock and are commonly used in food storage. To have an additional temperature point between cold-shock and optimum temperature (25 °C), 14 °C was selected. Finally, 28 °C was selected to be the heat-shock temperature, as psychrotrophic LAB are unable to grow at 30 °C or above [31].

## Results

### Bacterial growth

**Fig. 1:**
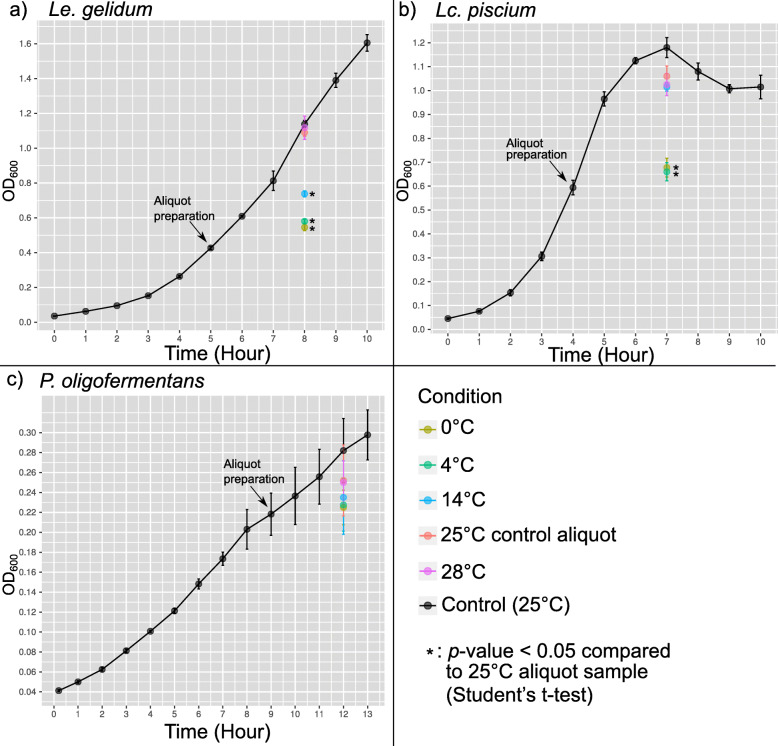
Growth curve of all three species based on optical density (OD_600_) values. The black colored points and line represent growth at 25 °C in liquid broth. Sampling times for aliquoting at different temperatures are marked in the figure with arrows. Colored points represent samples at different temperatures at 185 min; yellow: 0 °C, green: 4 °C, blue: 14 °C, red: 25 °C, and pink: 28 °C. Statistically significant (Student’s t-test *p*-value < 0.05) difference in growth compared to 25 °C control aliquot is indicated with an asterisk (*)

All bacteria were first grown at 25 °C and then aliquoted to five different temperatures for the specified time (see materials and methods; Fig. S1). *Le. gelidum* and *Lc. piscium* grew significantly (*p*-value < 0.05) slower at cold-shock temperatures (0 °C and 4 °C) compared to growth at control temperature (25 °C) (Fig. 1). At 14 °C, notably slower growth was observed only for *Le. gelidum*, indicating that *Le. gelidum* was more sensitive to the mild cold-shock temperature than the two other species. *P. oligofermentans* grew slightly slower at cold-shock temperatures (0 °C, 4 °C, and 14 °C) compared to growth in control temperature (25 °C), but the difference was not statistically significant. In addition, none of the species showed significant (*p*-value < 0.05) growth change at 28 °C compared to control temperature 25 °C (Fig. 1).

### Differentially expressed genes at different temperatures

**Fig. 2:**
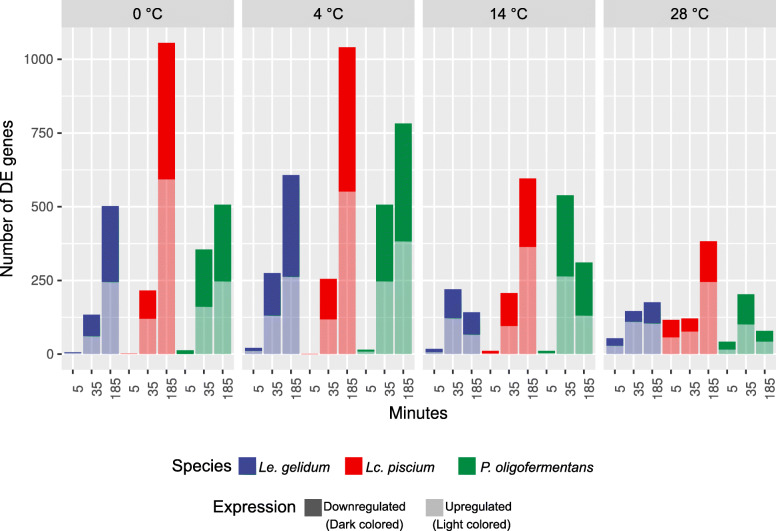
Number of differentially expressed genes of three species at 0 °C, 4 °C, 14 °C, and 28 °C relative to control temperature (25 °C). In general, the numbers of differentially expressed genes were low at the first timepoint but increased in the later timepoints. Blue bar represents *Le. gelidum,* red bar *Lc. piscium*, and green bar *P. oligofermentans*

Differential gene expression analysis showed that only a few genes were differentially expressed at the 5-min timepoint at cold-shock temperatures, indicating that 5 min was not sufficient to show a proper gene expression adaptation to cold temperatures in the species studied (Fig. 2). In contrast, a larger number of differentially expressed genes at 28 °C at the 5-min timepoint (Fig. 2) suggests that heat triggers a much faster and more robust change in gene expression than cold-shock treatment. The number of differentially expressed genes increased over time at 0 °C and 4 °C in all three species, while the number of differentially expressed genes decreased after the 35-min timepoint at 14 °C in *Le. gelidum* and *P. oligofermentans*, indicating that adaptation started after 35 min in these two species (Fig. 2). *Lc. piscium* had the highest number of differentially expressed genes in the conditions studied; about half of the genes were differentially expressed at 0 °C and 4 °C at the 185-min timepoint (Fig. 2).

**Fig. 3:**
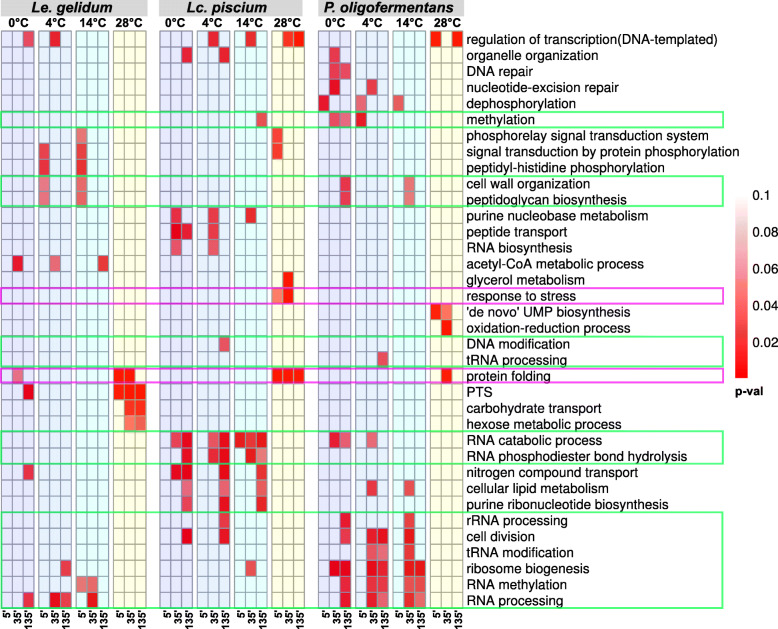
Heatmap of enriched GO terms of upregulated genes in *Le. gelidum, Lc. piscium*, and *P. oligofermentans.* Enriched GO terms of upregulated genes compared at different temperatures and timepoints. Ribosome, RNA processing, methylation, and cell-wall related terms are emphasized with a green box. Stress and protein-folding related terms that were enriched under heat-shock conditions are emphasized with a pink box. Comparisons were made against data from the 25 °C control. Red gradient represents the enrichment *p*-value, for which the scale is shown at the right side of the figure. Blue and yellow background colors were added to make cold and warm temperatures easily distinguishable. For simplification purposes, the figure does not include all enriched GO terms; all enriched terms are shown in Table S4

To classify the differentially expressed genes (Table S1, S2, S3), gene ontology (GO) enrichment analysis was performed. The results showed that RNA processing, ribosome biogenesis, and methylation (including DNA, rRNA, RNA, and tRNA methylation) GO terms were enriched for upregulated genes at cold temperatures in all species studied (Fig. 3). This suggests methylation, RNA processing, and ribosomal activities are common cold-shock responses in these species. Due to the low number of upregulated genes at the 5-min timepoint at cold temperatures, few enriched GO terms were observed at this time and only in *Le. gelidum*. Interestingly, the enriched terms were related to cell-wall and signaling, which implies that *Le. gelidum* sensed cold using signal transduction at a very early timepoint, and cell-wall related genes were first overexpressed at cold shock. In addition, cell-wall organization and peptidoglycan biosynthesis GO terms were enriched in *P. oligofermentans* for upregulated genes at late timepoints at cold temperatures. This indicates that cell-wall and membrane changes were part of a general cold-shock response. At 28 °C, upregulated genes were enriched for protein-folding GO terms in all studied species (Fig. 3). Interestingly, carbohydrate-metabolism related GO terms were also enriched for upregulated genes at 28 °C in *Le. gelidum*. For downregulated genes, enrichment of ATP synthesis-related GO terms was detected at cold temperatures in all three species, indicating slow growth (Table S4).

### Cold-shock, heat-shock, and stress-related genes

**Fig. 4:**
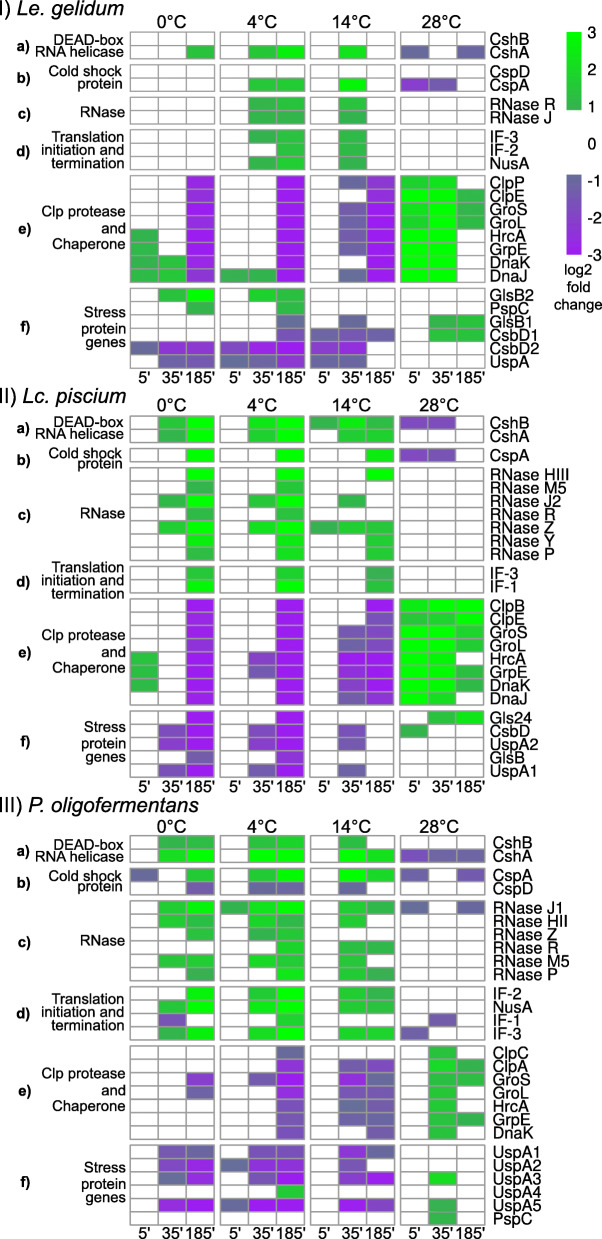
log_2_ fold-change heatmap of known cold- and heat-shock related genes in I) *Le. gelidum*, II) *Lc. piscium*, and III) *P. oligofermentans.* **a** DEAD-box RNA helicase genes, **b** cold-shock protein genes, **c** RNase genes, **d** translation initiation and termination genes, **e** Clp proteases and chaperones, and **f** stress protein genes. Comparisons were made against data from the 25 °C control. The log_2_ fold-change scale is shown at the right corner

We focused on known cold-shock response genes, such as cold-shock proteins, DEAD-box RNA helicases, and RNases*.* All three species harbored the cold-shock protein gene cspA, which was upregulated at cold temperatures and downregulated at 28 °C in all species (Fig. 4I(b), II(b), III(b)). In addition, *cspD* (a paralog of *cspA*) was also detected in *Le. gelidum* and *P. oligofermentans*. Interestingly, *cspD* was not upregulated in *Le. gelidum* and was downregulated in *P. oligofermentans* at cold temperatures (Fig. 4II(b)). While several RNase genes were upregulated at cold temperatures in *Lc. piscium* and *P. oligofermentans*, only two RNase genes were upregulated in *Le. gelidum* (Fig. 4I(c), II(c), III(c)). We also observed that DEAD-box RNA helicase genes were cold induced, since *cshA* was upregulated at cold temperatures in all studied species and *cshB* was upregulated in *Lc. piscium* and *P. oligofermentans*. The cold induced nusA-IF2 operon in *E. coli* [32] was present in all studied species*,* and it (*rimP*, *nusA*, *ylxR*, ribosomal protein L7AE gene, IF-2) was upregulated at cold temperatures in *Le. gelidum* and *P. oligofermentans*. In addition to the nusA-IF2 operon, upregulation of the translation initiation factor IF-3 was detected in all three species and upregulation of IF-1 in *Lc. piscium* and *P. oligofermentans* at cold temperatures (Fig. 4I(d), II(d), III(d)). Interestingly, none of the known cold-shock response genes were upregulated at 14 °C at the 185-min timepoint in *Le. gelidum*, although significant upregulation was seen at 35-min timepoint (Fig. 4I).

The heat-inducible transcription repressor *hrcA*, chaperone genes (*groS*, *groL*, *dnaK*, and *dnaJ*), Clp protease genes (*clpP*, *clpE*), and the chaperone-binding gene *grpE* were significantly upregulated at heat-shock temperature (28 °C) in all three species, with simultaneous downregulation of these genes at cold temperatures (Fig. 4I(e), 4II(e), 4III(e)). Upregulation of most heat-shock genes was not detected at the 185-min timepoint in *Le. gelidum* and *P. oligofermentans*.

Most of the stress-related genes were downregulated at cold temperatures in all species. We did not observe any upregulated stress genes at cold temperatures in *Le. piscium* (Fig. 4II(f)). Conversely, at least one stress-related gene was upregulated at 28 °C in all species (Fig. 4I(f), II(f), III(f)), indicating that heat creates a stronger stress reaction in the species studied.

### Pathway enrichment and changes of metabolism at different temperatures

**Fig. 5:**
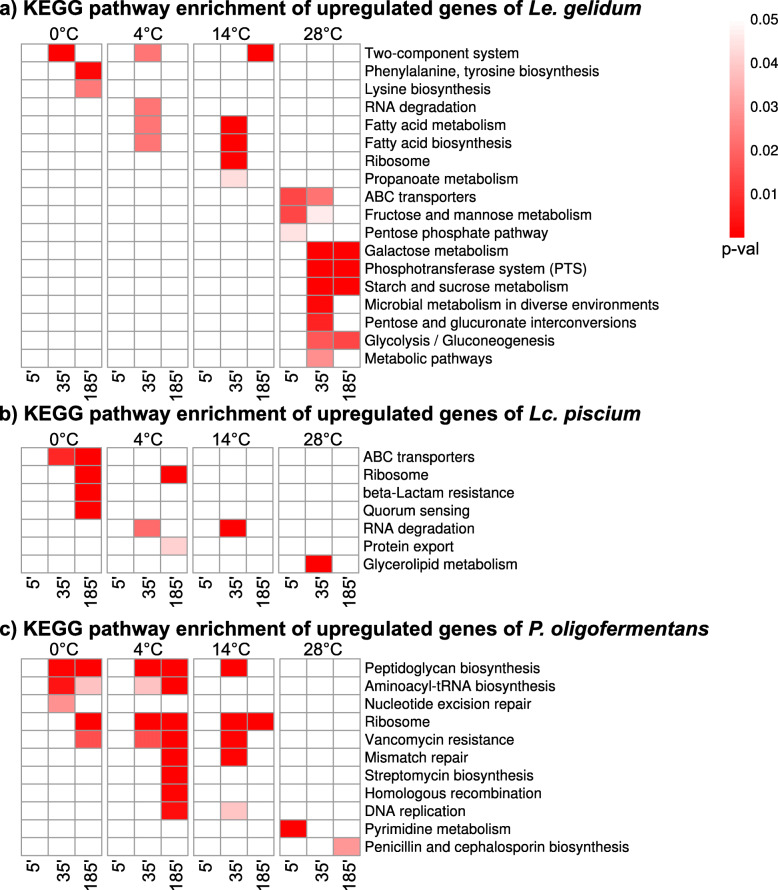
KEGG pathway enrichment for upregulated genes of **a**) *Le. gelidum*, **b**) *Lc. piscium*, and **c**) *P. oligofermentans*. Figure shows heatmap of enriched KEGG pathways for upregulated genes at different temperatures compared to 25 °C control. Enriched KEGG pathways are marked with red. Red gradient represents enrichment *p*-value, for which the scale is shown at the right corner

KEGG pathway enrichment analysis for upregulated genes showed that ribosome KEGG term was significantly (*p*-value < 0.05) enriched in all three species at cold temperatures, indicating that ribosome-related changes were a common cold-shock response (Fig. 5a, b, c). In addition, the two-component system KEGG term was enriched at all cold temperatures in *Le. gelidum* (Fig. 5a). It can be predicted that the two-component system is an important factor to sense cold in *Le. gelidum*. At cold temperatures, cell-wall and membrane-related KEGG terms, such as fatty acid biosynthesis, beta-lactam resistance, and peptidoglycan biosynthesis were enriched, indicating that cell-wall and membrane changes occurred in all three species (Fig. 5a, b, c). Enrichment of aminoacyl-tRNA biosynthesis KEGG term in *P. oligofermentans* at 0 °C and 4 °C suggests that production of aminoacyl-tRNA was part of the cold-shock response (Fig. 5c). In *Le. gelidum*, upregulated genes at 28 °C were mainly enriched for central metabolism KEGG terms, such as glycolysis, starch and sucrose metabolism, and galactose metabolism (Fig. 5a). Downregulated genes at cold temperatures were mostly enriched for central metabolism KEGG terms in all species, indicating metabolism was slower at cold temperatures (Fig. S2). Based on the metabolic pathway modelling and metabolic pathway enrichment for up- and downregulated genes (Fig. S3), citrate metabolism in *Le. gelidum* changes due to temperature; citrate metabolism genes were upregulated at cold temperatures and downregulated at 28 °C (Fig. S3a, d).

### Gene network inference

**Fig. 6:**
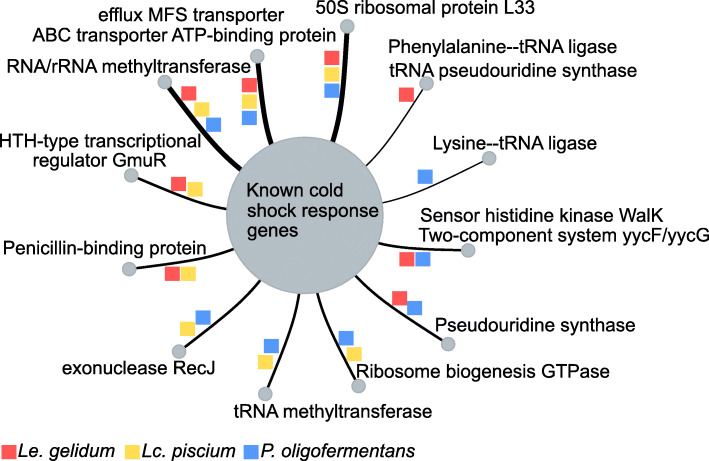
Summarized gene interactions for known cold-shock response genes. Figure shows genes that interacted with known cold-shock response genes in all three species. Interaction is represented with a line and color indicates species

To identify gene interactions and detect novel cold- and heat-shock response genes, we used a simple guilt-by-association approach by performing gene network inference analysis and gene interaction network-based clustering for all differentially expressed genes. The results showed that more than 80 clusters including at least two genes were identified in all three species (Table S5). Cold-shock response genes (*cspA*, *cshA*, RNases) were present either in the same cluster or clusters that were linked to each other (Fig. S4). Pseudouridine synthesis related genes and several methylation genes were found within the cold-shock related clusters in all species (Fig. 6), which indicates there is a strong interaction between these genes and suggests that methylation and pseudouridine plays a role in cold adaptation in all species studied. Similarly, ribosomal protein genes were linked to cold-shock response genes (Fig. 6), indicating they might play a role in cold adaptation. We observed that the two-component system regulatory protein genes *yycH* and *yycFG* were clustered with cold-shock response genes in *Le. gelidum* and *P. oligofermentans* (Fig. 6). In addition, the two-component sensor histidine kinase gene *hpk4* (CBL92274.1) in *Le. gelidum* and sensor histidine kinase (CEN29277.1) in *Lc. piscium* were linked to cold-shock response genes. This indicates that these sensors might play a role in cold sensing. Interestingly, DNA repair genes, such as *recA*, *recF*, and *recJ*, were clustered together with cold-shock response genes in *Lc. piscium*, suggesting DNA repair mechanisms are needed for cold adaptation.

All heat-shock related genes were clustered together in all three species and the number of the links was smaller compared to cold-shock response genes. As expected, the genes within the heat-shock clusters were significantly (*p*-value < 0.05) enriched for the protein-folding GO term, as most of the heat-shock genes were chaperones. Heat-shock related genes and the putative TetR family transcriptional regulator gene were clustered together in all three species, indicating the potential role of TetR in heat-shock gene regulation. In addition, there was a link between heat-shock genes and metal cation transporter genes in *Le. gelidum* (Fig. S4a).

### Transcription factor binding site prediction

We wanted to understand whether the genes clustered together by expression patterns would also be regulated with similar transcription factors. We first assessed whether any known transcription factor binding site motifs were enriched in the gene upstream regions of the three genomes studied. The result showed that CcpA, MalT, GalR, GalS, MtrB, Crp, and RpoD transcription factor binding sites occurred significantly (*p*-value < 0.05) commonly in all three species (Table S6). Since the cold-shock protein gene *cspA* can act as a transcription enhancer by binding to the 5′-ATTGG-3′ in the promoter regions of genes [33], we specifically searched for it and detected more than 280 upstream regions with the 5′-ATTGG-3′ motif (Table S7), including both cold- and heat-induced genes such as RNases, *cspA*, and *groS* (Table S7).

To predict de novo transcription binding sites, motif discovery analysis was performed for upstream regions of the upregulated genes for all conditions. Several motifs were discovered in all species (Table S8 [*Le. gelidum*], Table S9 [*Lc. piscium*], Table S10 [*P. oligofermentans*]). However, only a few of them were significantly (E-value < 0.05) similar to known motifs in transcription factor binding site (TFBS) databases. Motifs significantly (E-value < 0.05) similar to the CcpA binding site were discovered in the upstream regions of upregulated genes at 0 °C, 4 °C, and 28 °C in *Le. gelidum* (Table S8). In *Lc. piscium,* two of the discovered motifs were matched with a motif from TFBS database; at 14 °C at the 35-min timepoint, the motif matched with the PhoP motif from PRODORIC database [34] and at 28 °C at the 185-min timepoint with the rpoD17 motif from DPInteract database [35] (Table S9). A CtsR-binding site like motif was discovered in the upstream regions of upregulated genes at 28 °C at the 5-min timepoint in *Lc. piscium*, even though the de novo motif finding E-value score was not significant. Although database match analysis showed that some motifs in *P. oligofermentans* were significantly (E-value < 0.05) similar to the MalT motif from PRODORIC database [34], they were more likely Shine-Dalgarno sequence motifs of ribosomal binding sites (Table S10).

To more closely examine the co-expressed genes, clusters that were created using gene inference analysis were analyzed for de novo motif discovery. Motifs were discovered in cold-shock related clusters in *Lc. piscium* (Table S11, cluster 2) and *P. oligofermentans* (Table S12, cluster 4, 6, 7, 25, 32). However, neither of the discovered motifs were matched with any known transcription factor binding site motif. A motif with statistically significant E-value (< 0.05) was observed for a cluster of heat-shock related genes in *Le. gelidum* (Table S13, cluster3) and was significantly (E-value < 0.05) similar to HrcA motif in RegPrecise database [36]. Upstream regions of four heat-shock related genes (*clpE*, *groS*, *hrcA*, and *clpP*) and one hypothetical protein gene contributed to the construction of the motif (Table S13). Similarly, a CtsR-binding site like motif, but without significant E-value, was found for a cluster of heat-shock related genes in *Lc. piscium* (Table S11, cluster 3). In addition, GalR- and CcpA-binding site like motifs were discovered for several clusters of central metabolism related genes in both *Le. gelidum* (Table S13, cluster 4, 15) and *P. oligofermentans* (Table S12, cluster 2, 10, 28, 30).

### Activity of genes linked with food spoilage

**Fig. 7:**
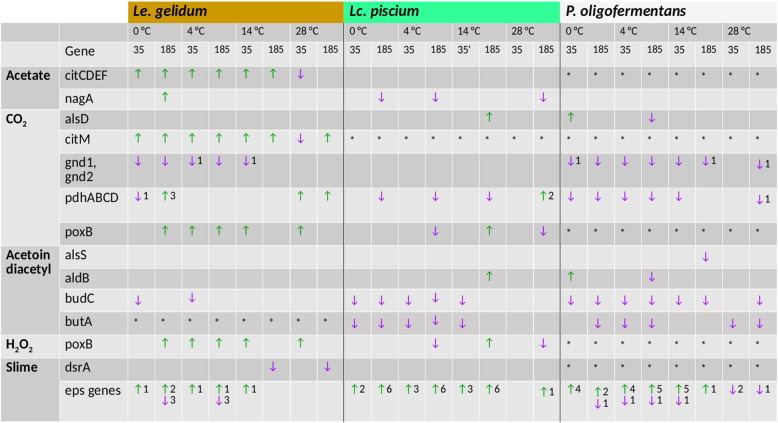
Expression reaction of spoilage-related genes at different temperatures in the three species. The data on genes related to formation of spoilage compounds in *Le. gelidum* and *Lc. piscium* have been presented previously [12, 14]. In this table, the expression-level changes of these genes and homologs at different temperatures are shown for all three species. Red arrows represent gene upregulation and blue arrows represent gene expression downregulation. The number next to the arrow indicates how many genes were up- or downregulated, if not all target genes were differentially expressed. Empty boxes indicate genes that were not differentially expressed. * indicates the homolog of the target gene was not found. The log_2_ fold-change values are listed in Table S14

We also specifically assessed the gene expression reaction of spoilage genes [12, 14] at both cold- and heat-shock temperatures in the three species. Spoilage-related genes were primarily upregulated at cold temperatures in *Le. gelidum* (Fig. 7, Table S14). In addition, slime-related eps genes were upregulated in both *Lc. piscium* and *P. oligofermentans* at cold temperatures.

### Validating RNA-Seq results with ddPCR

To validate the differential expression results obtained using RNA-seq data, we measured expression levels of eight selected genes (*mapA*, *nagB, pfl, fruK, infC, ftsQ, infB,* and 16S rRNA) using droplet digital PCR (ddPCR). Comparison of log_2_ fold changes of RNA-seq data and ddPCR data revealed significant correlation (Pearson’s correlation coefficient = 0.94) between the two methods (Fig. S5).

## Discussion

Cold-shock and heat-shock responses are both ancient systems and share features among bacteria and also to some extent with multicellular organisms. We were interested in the transcription-level reactions of *Leuconostoc gelidum*, *Lactococcus piscium*, and *Paucilactobacillus oligofermentans* to both cold and heat shock. The conducted experiments will help us understand how these reactions are organized at the genome level, thus also unravelling LAB function at temperatures relevant for food storage.

Although bacteria can harbor several cold-shock protein (CSP) gene paralogues, not all CSP genes are cold induced. For example, only four (*cspA*, *cspB*, *cspG*, and *cspI*) of the total nine CSP genes are cold induced in *E. coli* [37]*.* Similarly, it has been shown that four (*cspA*, *cspB*, *cspC*, and *cspD*) of the total five CSP genes are cold induced in mesophilic lactic acid bacteria *Lactococcus lactis* [38]. We observed that *Le. gelidum* and *P. oligofermentans* harbored two CSP genes (*cspA, cspD*). Interestingly, *Lc. piscium* harbored only one CSP gene (*cspA*). In all three species, only *cspA* was cold induced, i.e. significant (*p*-adj < 0.05) upregulation was observed at cold temperatures based on RNA-seq. This indicates that *cspA* was the main responsible CSP gene in these species for cold adaptation, while *cspD* genes may not play a role in cold adaptation in *Le. gelidum* and *P. oligofermentans*. DEAD-box RNA helicase genes participate in degradation and unwinding of RNA and ribosome biogenesis during cold shock [39]. Our RNA-seq results revealed that DEAD-box RNA helicase genes (*cshA* in *Le. gelidum*, *cshA* and *cshB* in *Lc. piscium* and *P. oligofermentans*) were one of the most significantly upregulated genes in all three species at cold temperatures (Table S1, S2, S3). This suggests that DEAD-box RNA helicase genes have a critical role in these three species at cold temperatures. In addition to DEAD-box RNA helicase, we observed that RNase R and RNase J were the only cold induced RNase genes in *Le. gelidum*, while several RNase genes were cold induced in *Lc. piscium* and *P. oligofermentans* (Fig. 4II c, III c). Although RNase R was reported as the major RNase gene in *E. coli* [24], upregulation of several RNase genes in *Lc. piscium* and *P. oligofermentans* indicates that other RNase genes also participate in cold adaptation.

Translation regulation through translation initiation factors (IF-1, IF-2, IF-3) at cold temperatures has been shown by previous studies [40]. In particular, IF-3 significantly favors translation of CSPs *in E. coli* [41]. In all three species, the IF-3 gene was significantly upregulated at cold temperatures, indicating similar translation regulation in LAB. In addition, IF-2 has been shown to be located in the same operon with nusA in *E. coli*, and the nusA-IF-2 operon affects ribosome maturation at cold temperatures [32]. We observed that in all three species, IF-2 was also in the same operon with *nusA*. The significant upregulation of nusA-IF-2 in *Le. gelidum* and *P. oligofermentans* at cold temperatures indicates that the nusA-IF-2 operon may be important for cold adaptation in these species. However, no upregulation of the nusA-IF-2 operon was observed in *Lc. piscium.*

In addition to commonly known cold-shock response genes, we also identified several other cold induced genes in all three species during cold-shock treatment. The functions of these genes were summarized using GO enrichment and KEGG enrichment analysis (Figs. 3 and 5). Ribosome biogenesis, methylation, RNA processing/RNA catabolic processes, and fatty acid/lipid metabolism GO and KEGG terms were enriched for upregulated genes at cold temperatures, indicating a similar reaction to cold by the three species. Metabolic pathway enrichment analysis clearly showed that central metabolism genes were downregulated at cold-shock temperatures in all species (Fig. S3). Citrate-metabolism genes were upregulated at cold-shock temperatures in *Le. gelidum* (Fig. S3), indicating that *Le. gelidum* favored the usage of citrate as a carbon source at cold-shock temperatures. In addition, we observed that some sugar-degradation genes, such as sucrose, maltose, and galactose degradation genes (Fig. S3) were upregulated at 28 °C. Therefore, we can predict that *Le. gelidum* switches its carbon source based on temperature. We did not observe such clear differences of carbon source usage based on temperature in *Lc. piscium* and *P. oligofermentans*. The upregulated metabolic pathway genes at cold-shock temperatures were related to cell-wall and cell membrane in these species (Fig. S3).

Not all significantly differentially expressed genes must be related to cold-shock response, since growth was also significantly (*p*-value < 0.05) affected by temperature change (Fig. 1). A large number of differentially expressed genes in *Lc. piscium* may be partially explained by different growth phases as shown in our previous transcriptomics study [14]. To directly identify the temperature change shock response genes, we took advantage of our complex study design (time-series study with several different conditions). This provided an opportunity to perform not only differential expression gene analysis but also gene network inference analysis and clustering based on gene expression profiles [42]. Clustered genes and linked clusters by gene network are expected to be functionally related [43–45], and inspecting clusters allowed us to predict genes directly related to the temperature change response.

Gene network inference and clustering results suggests that ribosomal protein and RNA methyltransferase genes were part of the cold-shock response in all three species, since the 50S ribosomal protein L33 gene, RNA/rRNA methyltransferase genes, and the ABC transporter ATP-binding protein gene were clustered with known cold-shock response genes (Fig. 6, Table S5).

Specifically, one ABC transporter gene (CBL90879.1, CEN27266.1, CUS25589.1 in *Le. gelidum*, *Lc. piscium*, and *P. oligofermentans*, respectively) and one multidrug efflux MFS transporter gene (CBL92085.1, CEN28822.1, CUS25692.1 in *Le. gelidum, Lc. piscium*, and *P. oligofermentans*, respectively) were clustered with cold-shock response genes and significantly upregulated during cold-shock treatment in all three species, indicating that functionality of these genes was also related to cold-shock response. Both ABC and MFS transporters can play a role in stress resistance, especially in antibiotic resistance [46]. However, previous studies related to psychrophilic organisms also support that ABC and MFS transporters play a role in cold adaptation [47, 48]. Upregulation of the multidrug ABC transporter gene at cold temperatures was reported for psychrophilic gram-negative bacteria *Flavobacterium psychrophilum* [47]. Similarly, upregulation of an MFS transporter gene at cold temperatures was observed in the psychrophilic fungus *Mrakia psychrophila* [48].

Ribosomal RNA methylation as a response to stress conditions has been previously observed. For example, deletion of *ksgA* (encoding RNA small subunit methyltransferase A) in *E. coli* causes cold sensitivity and changes in RNA biogenesis [49]. A large number of upregulated RNA methyltransferase genes at cold temperatures and clustering with cold-shock response genes revealed that RNA methylation plays a role in cold adaptation of the three species studied as a part of post-transcriptional regulation.

Although an interaction between tRNA methyltransferase and cold-shock genes was only detected in *Lc. piscium* and *P. oligofermentans,* tRNA modification gene, tRNA pseudouridine synthase, and tRNA ligase genes also clustered with cold-shock genes in *Le. gelidum* (Fig. 6). This indicated that tRNA modification was part of cold adaptation in all three species. A tRNAome study showed that tRNA abundance and tRNA modification significantly changed under stress in *Lc. lactis* [50]. It is interesting that tRNA modification is required for cold adaptation in extreme-thermophilic bacteria [51]. In addition, it has been shown that tRNA modification during environmental stress is critical [52]. The gene *trmD* (encoding guanine-N (1)-methyltransferase) is required for multi-drug resistance in *E. coli*, since it provides a control mechanism for translation of membrane proteins [52]. It is possible that tRNA modification in the three lactic acid bacteria studied provides a similar control system for cold-shock response genes, promoting their translation.

Two-component sensor histidine kinase activity of DesK-DesR at cold temperatures has been shown previously in *Bacillus subtilis* [53]. A homolog of DesK-DesR was not found in the species studied. However, several other sensor genes were upregulated during cold shock in all three species (Fig. S6). Genes that encode the two-component system WalR (*yycF*) and sensor kinase WalK (*yycG*) had similar expression profiles with cold-shock response genes in *Le. gelidum* and *P. oligofermentans*, implying that these sensor kinase genes may be linked to cold-shock response in both species. Previously, the sensor kinase WalK has not been reported as a temperature sensor, but it has been shown to have a role in regulating cell-wall modifications and peptidoglycan biosynthesis [54]. We suggest that *Le. gelidum* and *P. oligofermentans* use WalK to regulate cell-wall changes to adjust for cold temperature. Interestingly, the homologs of these genes (*walK*-*walR*) in *Lc. piscium* were not upregulated at cold temperatures. In addition, the *hpk4* gene (CBL92274.1), which encodes a two-component sensor histidine kinase, was clustered with cold-shock response genes in *Le. gelidum* and was one of the few upregulated genes at early timepoints at cold temperatures (Fig. S6, Table S1). Thus, we believe that *hpk4* is a potential cold-sensor gene and could play a role in regulation of cold-shock response genes in *Le. gelidum*.

In all three species, well known heat-shock response genes, such as chaperone genes, Clp protease genes, and the heat-inducible transcriptional repressor *hrcA* [26], were significantly upregulated at 28 °C (Fig. 4I e, II e, III e). The main activities of heat-shock response genes are to maintain the proper protein-folding mechanism in cells [26]. Unsurprisingly, protein folding was the only common GO term enriched for upregulated genes in all three species at 28 °C. As well as protein-folding, central metabolism GO terms were enriched in *Le. gelidum* for upregulated genes at 28 °C, indicating that central metabolism benefited from the temperature increase. We also observed that glycerol and glycerololipid metabolism-related genes were upregulated at 28 °C in *Lc. piscium*. It is likely that one of the heat-shock responses in *Lc. piscium* was to change formation of glycerol-based membrane lipids [55]. Notably, a large number of genes clustered with known cold-shock response genes, potentially signifying the difference in cold-shock adaptation mechanisms among different LAB species. However, heat-shock adaptation mechanisms seem to be highly conservative in LAB and were provided by only the known heat-shock response genes. We also noticed that the *ctsR* gene, a negative regulator of heat-shock Clp-family gene expression [56], was not harbored by *Le. gelidum* but was upregulated at heat-shock temperature in *Lc. piscium* and *P. oligofermentans.* Since *ctsR* is a negative regulator of Clp genes and these genes are required for heat-shock adaptation [56], the lack of the *ctsR* gene might be an advantage for *Le. gelidum* at heat-shock temperatures.

The LAB species studied here are found in cold-stored MAP food products and are the main driver of spoilage in these food products [3, 4]. Therefore, we were interested in the gene expression reactions of spoilage-related genes [12, 14] at different temperatures, especially at cold temperatures. We showed that most of the spoilage-related genes were upregulated at cold temperatures in *Le. gelidum*. In addition, slime-related genes were upregulated at cold temperatures in *Lc. piscium* and *P. oligofermentans*. Thus, we predicted that the level of spoilage activities at cold temperatures were higher for these species compared to 25 °C. We did not observe a clear trend for expression of spoilage genes at heat-shock (28 °C). However, upregulated metabolism-related genes in *Le. gelidum* indicated increased spoilage activities at 28 °C.

We were also specifically interested in transcription factor binding site motifs. We showed that the CcpA binding site motif was enriched for upregulated genes at both cold- and heat-shock temperatures. It has been shown that the CcpA regulon controls carbon metabolism in bacteria [57]. We observed that expression of carbon-metabolism genes was significantly affected by temperature change in *Le. gelidum*. Therefore, enrichment of the CcpA motif could be expected for upregulated genes at both cold and warm temperatures. In addition, we discovered a motif in the upstream regions of heat-shock genes in *Le. Gelidum* that was significantly (E-value < 0.05) similar to the HrcA binding site from RegPrecise database [36]. Similarly, analysis of upstream regions for the heat-shock genes in *Lc. piscium* allowed us to predict the binding site motif for heat-shock genes, which was similar to the CtsR binding site from RegPrecise database [36]. The motif discovery analysis did not show significant motifs for heat-shock genes in *P. oligofermentans.* However, we detected motifs for carbon-metabolism clusters, which were significantly similar to the CcpA binding site motif. This shows that *ccpA* plays a role in metabolism response to temperature change, similar to *Le. gelidum.*

## Conclusions

Here, we profiled gene expression changes of three psychrotrophic LAB at three timepoints and five different temperatures. The results provided an important understanding of the cold- and heat-shock adaptation mechanisms in psychrotrophic LAB. In addition to known cold-shock response genes, we highlighted the importance of rRNA and tRNA modification during cold-shock response in psychrotrophic LAB as a part of post-transcriptional regulation. We showed that ABC and efflux MFS transporter genes were involved in cold-shock response. These results might yield new directions for cold-shock studies in psychrotrophic organisms. Transcriptomic profiles during heat-shock treatment revealed that protein folding was the dominant reaction for psychrotrophic LAB. Upregulation of spoilage-related genes at cold temperatures suggests the level of spoilage activities was higher at cold temperatures, especially for *Le. gelidum*. Further gene inference analysis revealed several candidate genes involved in cold- and heat-shock adaptation and sensing. The candidate genes identified using gene inference analysis will be beneficial for further studies. Finally, we identified binding-site motifs for heat-shock genes in *Le. gelidum* and *Lc. piscium* using de novo motif search analysis.

## Methods

### Experimental setup and sampling

Three psychrotrophic LAB species, *Leuconostoc gelidum* subsp. *gasicomitatum* LMG 18811, *Lactococcus piscium* MKFS47, and *Paucilactobacillus oligofermentans* DSM 15707, were used for the experiment. A frozen glycerol stock solution (1.8 ml) of a bacterium was inoculated into 9 ml of de Man-Rogosa-Sharpe medium without acetate (MRS-Ac) and incubated overnight at 25 °C. One microliter of the culture was streaked on a MRS-Ac agar plate and grown in anaerobic conditions over three nights at 25 °C. One colony was then transferred to 9 ml of MRS-Ac medium. After 17 h (for *Le. gelidum* and *Lc. piscium*) or 31 h (for *P. oligofermentans*), a 100-μl aliquot was again inoculated into 9 ml of MRS-Ac medium and grown for a further 12 h (for *Le. gelidum* and *Lc. piscium*) or 24 h (for *P. oligofermentans*). An aliquot of 90 μl (*Le. gelidum* and *Lc. piscium*) or 80 μl (*P. oligofermentans*) of these precultures was inoculated into 100 ml MRS-Ac and grown over one night (*Le. gelidum* and *Lc. piscium*) or over two nights (*P. oligofermentans*) at 25 °C until an OD_600_ value of approximately 0.4. The cultures were then diluted 1:10 to a final OD_600_ value 0.04 in a total volume of 100 ml. The obtained cultures were grown at 25 °C in four replicates. The growth of these cultures was followed by performing OD_600_ measurements every hour for 10 h (*Le. gelidum* and *Lc. piscium*) or 13 h (*P. oligofermentans*). After 4 h (*Lc. piscium*), 5 h (*Le. gelidum*), and 9 h (*P. oligofermentans*) of growth (possibly exponential phase for *Lc. pisicum*, early exponential phase for *Le. gelidum*, and late lag phase for *P. oligofermentans* (Fig. 1)), 10-ml aliquots of each culture were transferred to five different temperatures (0 °C, 4 °C, 14 °C, 25 °C, and 28 °C). The temperatures were selected to include cold-shock temperatures (0 °C, 4 °C), mild cold-shock temperature (14 °C), and a heat-shock temperature (28 °C); 25 °C was used as the control temperature. For each bacterium, samples for RNA extraction were collected from the 100-ml bottles before aliquoting (timepoint 0 min) and from aliquots taken at different temperatures after 5 min, 35 min, and 185 min of growth (Fig. S1). Sample volumes were determined using the following formula: V = (1/1.5*OD_600_). The same sample volume was used at the 0-, 5-, and 35-min timepoints. Before sampling at 185 min, an OD_600_ measurement was performed to adjust the sample volume. Samples were immediately mixed with a 1/10 volume of cold ethanol-phenol mixture (10:1) to stop RNase activity. Cells were pelleted by centrifugation at 5000 x g for 3 min, frozen in liquid nitrogen, and stored in − 80 °C. Altogether, 192 samples were collected during the growth experiment.

### RNA extraction and transcriptome sequencing

RNA extraction was performed with a Nucleospin RNA kit (Macherey-Nagel, Düren, Germany) according to the manufacturer’s instructions with some modifications in the cell lysis step. Cells were resuspended in 700 μl of buffer RA1 supplemented with 7 μl of β-mercaptoethanol and then mechanically disrupted using Lysing Matrix E or B tubes (MP Biomedicals, Irvine, CA, USA) and FastPrep 24 tissue homogenizer (MP Biomedicals) at 5.5 m/s for 40 s. Quality and quantity of RNA extractions were analyzed using an Agilent 2100 Bioanalyzer and RNA 6000 Nano kit (Agilent, Santa Clara, CA, USA).

Ribosomal RNA removal was performed with Ribo-Zero rRNA removal reagent for bacteria (Illumina, San Diego, CA, USA) according to the manufacturer’s instructions using 1000 ng of total RNA and a 1/3 volume of kit solutions. rRNA-depleted RNA was purified with RNA Clean&Concentrator − 5 kit (Zymo Research, Irvine, California, USA) according to manufacturer’s instructions and eluted in 11 μl of RNA-free water.

QIAseq™ Stranded Total RNA Lib kit (Qiagen, Hilden, Germany) was used for RNA-seq library preparation according to manufacturer’s instructions with some modifications. A one-third volume of kit solutions was used together with 9.7 μl rRNA-depleted RNA. In the strand-specific ligation step, 0.3 μM truncated Truseq adapter was used instead of the adapter provided in the kit. Libraries were amplified using half (10 μl) of the purified ligation product in 1 x HF buffer with 0.2 mM dNTPs, 0.6 μM selected [58] dual-index primer, and one unit of Phusion Hot Start II High-Fidelity DNA polymerase (Thermo Fisher Scientific, Waltham, Massachusetts, United States) in a total volume of 50 μl. The PCR protocol was 98 °C, 30 s; 18 x (98 °C, 10 s; 65 °C, 30 s; 72 °C, 10 s); 72 °C, 5 min. Concentrations of amplified libraries were measured with a Qubit fluorometer and dsDNA HS assay kit (Invitrogen, Waltham, MA, USA). Size distributions were visualized with Fragment Analyzer and High Sensitivity NGS Fragment Analysis kit (Advanced Analytical, Parkersburg, WV, USA). After pooling, libraries were concentrated using Amicon Ultra 100 K columns (Millipore, Burlington, MA, USA) and purified twice with 0.9 x AMPure XP beads (Beckman Coulter, Brea, CA, USA). For *Le. gelidum* and *P. oligofermentans* libraries, size selection of 300 to 600 bp fragments was performed using BluePippin and 2% agarose gel cassette (Sage Science, Beverly, MA, USA). PEG (8–8.5%) size selection was used with *Lc. piscium* libraries. Illumina NextSeq 500 was used to sequence the single-end 75 bp RNA-seq libraries.

### Sequencing data processing

Adaptor trimming and quality filtering of the reads was performed using Trimmomatic v0.36 [59] using these parameters “TruSeq3-SE.fa:2:30:10 SLIDINGWINDOW:3:20 HEADCROP:10 MINLEN:30”. SortMeRNA v2.1 [60] was used for ribosomal RNA filtering. Filtered reads were mapped to genomes using Bowtie2 v2.3.4.3 [61] and were sorted using Samtools v1.8 [62]. Read counts for genes were produced using HTSeq v0.11.0 [63] with union mode. DESeq2 v1.22.2 R package [64] was used for differential gene expression analysis. The significance threshold for differential gene expression was set for *p-*adjusted ≤ 0.05 and |log_2_FoldChange| ≥ 1. PANNZER2 [65] was used for the functional and GO term annotation. The enrichment analysis for GO terms was performed using topGO v2.36.0 R package [66], the enrichment threshold was set for *p*-value ≤ 0.05. KEGG pathway enrichment was performed using enrichKEGG function of clusterProfiler v3.14.3 R package [67]. KEGG annotation of *Le. gelidum* and *Lc. piscium* was obtained from the KEGG database [68]. Since *P. oligofermentans* was not found in the KEGG database, KAAS (KEGG Automatic Annotation Server) web server [69] was used to create KEGG annotation and KEGG pathways of *P. oligofermentans*. We also used Pathway Tools v19.0 [70] for metabolic pathway model construction and metabolic pathway enrichment.

### Gene network inference analysis and motif discovery

Gene network inference was performed using seidr toolkit [42] using the following 11 gene network inference methods: CLR, GENIE3, Aracne2, Pearson, Spearman, NARROMI, TIGRESS, PCor, PLSNET, llr-ensemble, and el-ensemble. The results were aggregated into a network using seidr toolkit. Hard threshold was chosen following the recommendations from the developer. The network clustered using Infomap [71] and clusters were visualized using the Map generator applet from MapEquation [72].

For the motif-based sequence analysis, MEME suite v5.0.5 [73] was used. The upstream regions had a minimal length of 50 bp and up to 300 bp were extracted using python script (https://github.com/peterthorpe5/intergenic_regions). Motif discovery of upstream regions was performed using MEME and discovered motifs were searched against transcription factor binding site databases, such as CollecTF [74], PRODORIC [34], RegTransBase [75], RegPrecise [36], DPINTERACT [35], and Swiss Regulon [76] using Tomtom [77]. The listed databases were downloaded from the MEME suite as meme database format, except RegPrecise. For RegPrecise, all transcription factor binding site motifs for *Lactobacillaceae* were downloaded and converted to meme database using sites2meme script from MEME suite v5.0.5 [73]. Ame [78] was used to test whether motifs in transcription binding site databases were enriched in upstream regions of our reference genomes.

### ddPCR validation

Three out of four replicate samples grown at 4 °C, 14 °C, 25 °C, and 28 °C and collected at 185 min were analyzed by ddPCR to verify the RNA-seq results. The primers and the protocol were previously published by Andreevskaya et al. [16]. *For Le. gelidum*, expression of *mapA*, *nagB*, *ftsQ*, *infB*, and 16S rRNA genes, for *Lc. piscium*, expression of *pfl*, *fruK*, *ftsQ*, *infB*, and 16S rRNA genes, and for *P. oligofermentans*, expression of *ccpA*, surf, *infC*, *ftsQ*, *infB*, and 16S rRNA genes were analyzed. For each species, the reference genes used for normalization of gene copy numbers were those that showed the most stable expression levels (according to RNA-seq) among the samples studied: *infB, ftsQ,* and 16S rRNA gene (*Le. gelidum* and *Lc. piscium*), or *ccpA*, surf, and 16S rRNA gene (*P. oligofermentans*).

## Supplementary Information

**Additional file 1: Figure S1.** Experimental setup and sampling summary. Each of the three species were grown at 25 °C as four replicates. After collecting the first sample (control; timepoint 0 min), aliquots were taken at five temperatures (0 °C, 4 °C, 14 °C, 25 °C, and 28 °C). For each aliquot, samples were collected after 5 min, 35 min, and 185 min.

**Additional file 2: Table S1.** List of differentially expressed genes in *Le. gelidum.*

**Additional file 3: Table S2*.*** List of differentially expressed genes in *Lc. piscium.*

**Additional file 4: Table S3*.*** List of differentially expressed genes in *P. oligofermentans.*

**Additional file 5: Table S4*.*** List of enrichment GO terms for up- and downregulated genes in *Le. gelidum, Lc. piscium, P. oligofermentans.*

**Additional file 6: Figure S2.** KEGG pathway enrichment for downregulated genes of *a) Le. gelidum, b) Lc. piscium, c) P. oligofermentans****.*** Figure shows heatmap of enriched KEGG pathways for downregulated genes at different temperatures compared to 25 °C control. Enriched KEGG pathways are marked with red. Red gradient represents enrichment *p*-value, for which scale is shown at the right corner.

**Additional file 7: Figure S3.** Metabolic pathway enrichment for upregulated genes of a) *Le. gelidum*, b) *Lc. piscium*, c) *P. oligofermentans* and downregulated genes of d) *Le. gelidum*, e) *Lc. piscium*, and f) *P. oligofermentans*. Figure shows heatmap of enriched metabolic pathways for up- and downregulated genes at different temperatures compared to 25 °C control. Enriched metabolic pathways are marked with red. Red gradient represents enrichment *p*-value, for which scale is shown at the right corner. The metabolic pathway modelling and metabolic pathway enrichment analysis was performed using Pathway Tools.

**Additional file 8: Table S5.** List of gene clusters based on gene network inference analysis in *Le. gelidum*, *Lc. piscium*, and *P. oligofermentans*.

**Additional file 9: Figure S4.** Clustered genes based on gene network inference in a) *Le. gelidum*, b) *Lc. piscium*, and c) *P. oligofermentans*. Each node represents a cluster and edges represent predicted links between clusters. Number of genes within the cluster is shown in the center of the node. Below the cluster number (Cl), the top-scored enriched GO term is given. Clusters including cold-shock and heat-shock response genes are marked in the figure. Genes within the clusters are listed in Table S5. The scales used are described in the box. For simplification, the figure shows only the top 20 links with the highest weight and the associated connected clusters.

**Additional file 10: Table S6.** List of known motifs enriched within upstream regions of all genes in *Le. gelidum*, *Lc. piscium*, and *P. oligofermentans* based on analysis of motif enrichment.

**Additional file 11: Table S7.** List of genes with a CspA binding site motif in their upstream regions in *Le. gelidum*, *Lc. piscium*, and *P. oligofermentans*.

**Additional file 12: Table S8.** List of motifs with significant E-value discovered based on upstream regions of upregulated genes in *Le. gelidum*.

**Additional file 13: Table S9.** List of motifs with significant E-value discovered based on upstream regions of upregulated genes in *Lc. piscium*.

**Additional file 14: Table S10.** List of motifs with significant E-value discovered based on upstream regions of upregulated genes in *P. oligofermentans*.

**Additional file 15: Table S11.** List of motifs with significant E-value discovered based on upstream regions of clustered genes in *Lc. piscium*.

**Additional file 16: Table S12.** List of motifs with significant E-value discovered based on upstream regions of clustered genes in *P. oligofermentans*.

**Additional file 17: Table S13.** List of motifs with significant E-value discovered based on upstream regions of clustered genes in *Le. gelidum*.

**Additional file 18: Table S14.** List of spoilage-related genes and log_2_ fold changes in the three species.

**Additional file 19: Figure S5.** Comparison of relative expression changes (log_2_ fold change) for selected genes obtained using ddPCR versus RNA sequencing (RNA-seq). Samples from 185 min were used for the comparison. Temperatures are mentioned after a gene name. A letter in parentheses after a gene name represents the source organism (G, P, and O represents *Le. gelidum*, *Lc. piscium*, and *P. oligofermentans*, respectively). Genes of *Le. gelidum*; *mapA* (LEGAS_1151) and *nagB* (LEGAS_1624) and genes of *Lc. piscium*; *pfl* (LACPI_1736) and *fruK* (LACPI_2020) were normalized using concentration of the housekeeping gene *infB*. The genes of *P. oligofermentans*; *infC* (LACOL_0746), *ftsQ* (LACOL_1184), *infB* (LACOL_1061) were normalized using concentration of the 16S rRNA gene.

**Additional file 20: Figure S6.** log_2_ fold-change heatmap of sensing/signal-related genes in all three species. The log_2_ fold-change scale is indicated in the right corner.

## Data Availability

All sequencing data have been deposited in the European Nucleotide Archive (ENA) under accession code PRJEB38386.

## Abbreviations

CSP: Cold-shock protein
LAB: Lactic acid bacteria
RNase: Ribonuclease
GO: Gene Ontology
Cl: Cluster
TFBS: Transcription factor binding site
KEGG: Kyoto Encyclopedia of Genes and Genomes
ddPCR: Droplet digital PCR
IF: Initiation factor
