# Supplementary figures and images for: Transcriptomic time-series analysis of cold- and heat-shock response in psychrotrophic lactic acid bacteria
Source: BMC Genomics. 2021 Jan 7;22:28. doi: 10.1186/s12864-020-07338-8 (PMC7788899; doi:10.1186/s12864-020-07338-8)

aliquots to selected temperatures

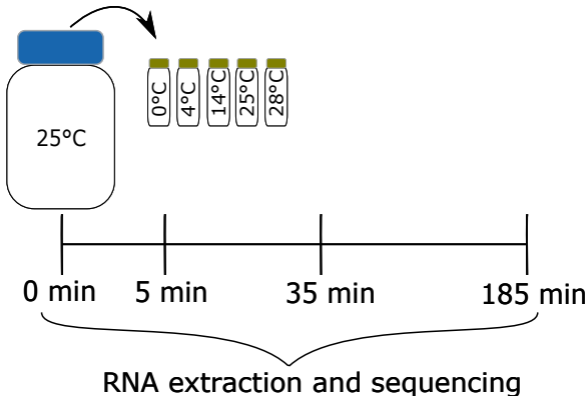

Supplement: Supplementary file 1 — Additional file 1: Figure S1. Experimental setup and sampling summary. Each of the three species were grown at 25 °C as four replicates. After collecting the first sample (control; timepoint 0 min), aliquots were taken at five temperatures (0 °C, 4 °C, 14 °C, 25 °C, and 28 °C). For each aliquot, samples were collected after 5 min, 35 min, and 185 min. [file 12864_2020_7338_MOESM1_ESM.pdf]

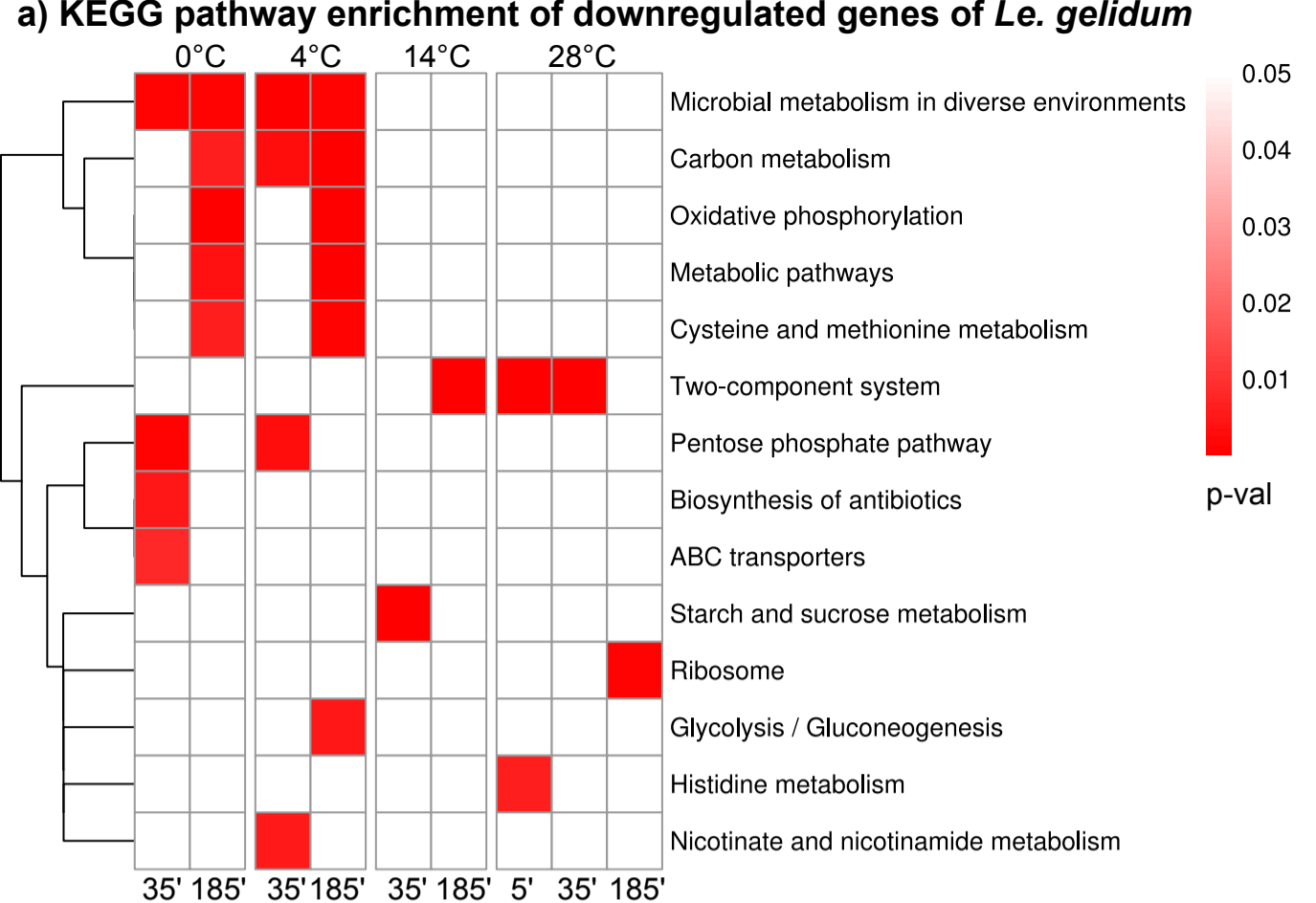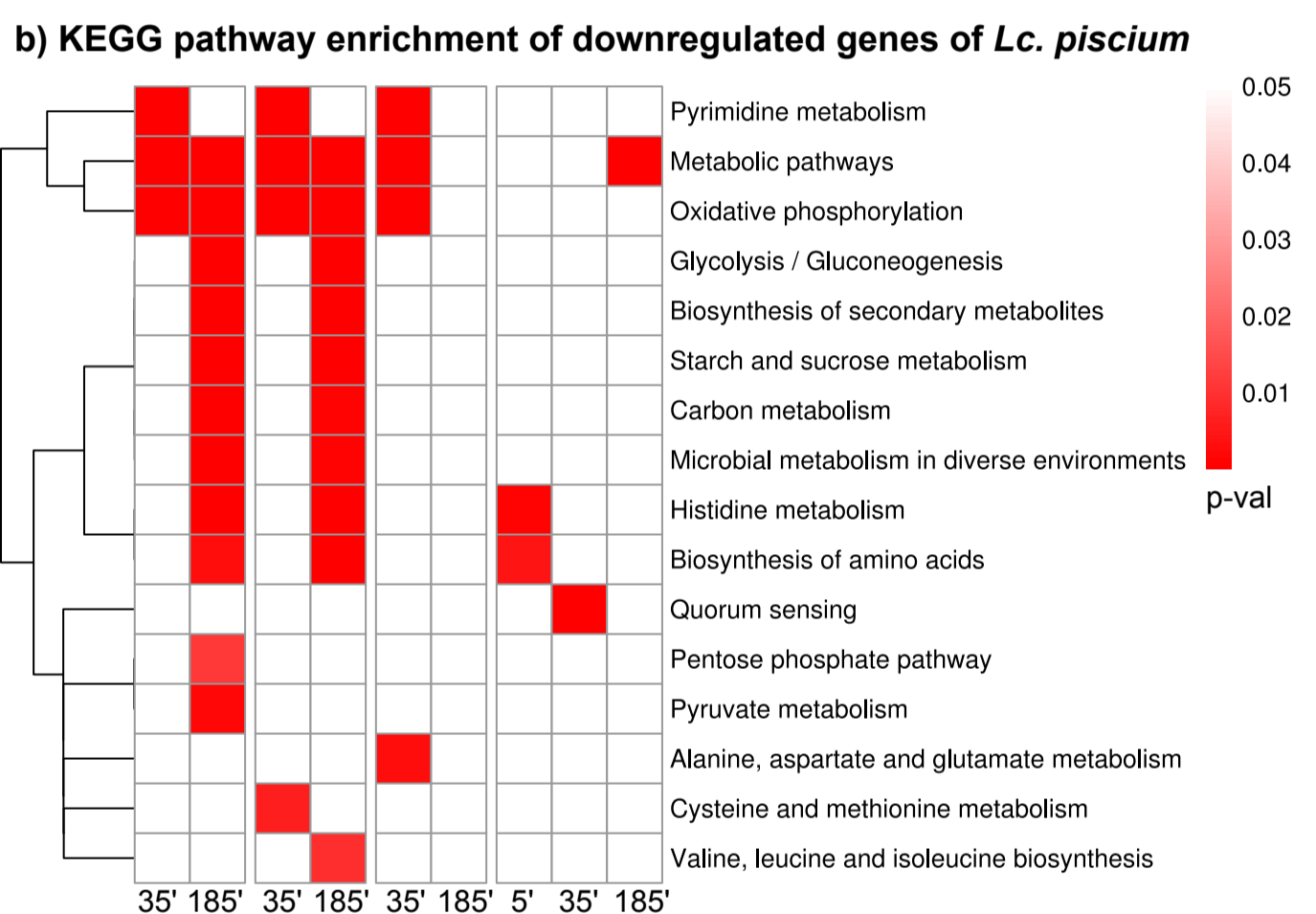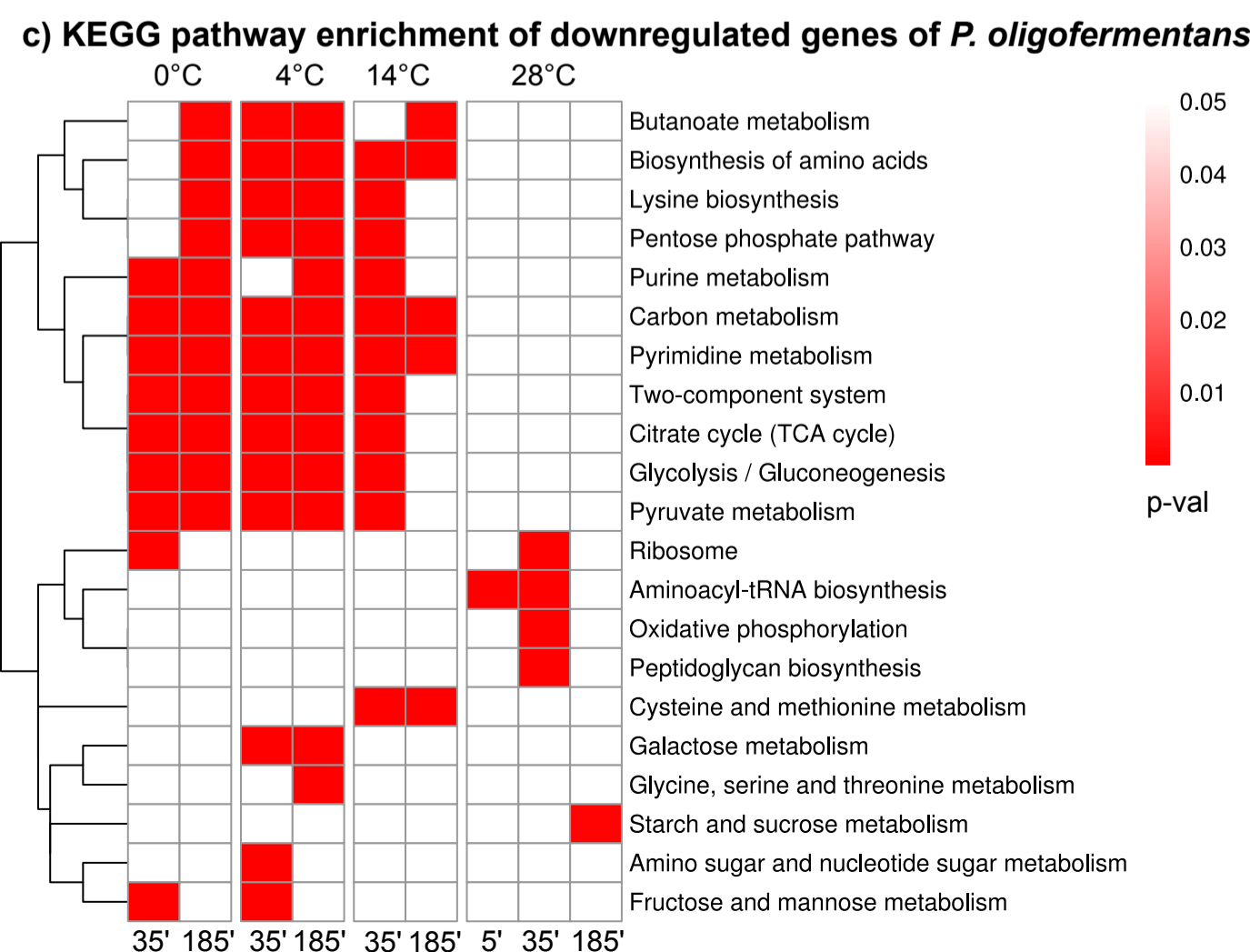

Supplement: Supplementary file 6 — Additional file 6: Figure S2. KEGG pathway enrichment for downregulated genes of a) Le. gelidum, b) Lc. piscium, c) P. oligofermentans. Figure shows heatmap of enriched KEGG pathways for downregulated genes at different temperatures compared to 25 °C control. Enriched KEGG pathways are marked with red. Red gradient represents enrichment p-value, for which scale is shown at the right corner. [file 12864_2020_7338_MOESM6_ESM.pdf]

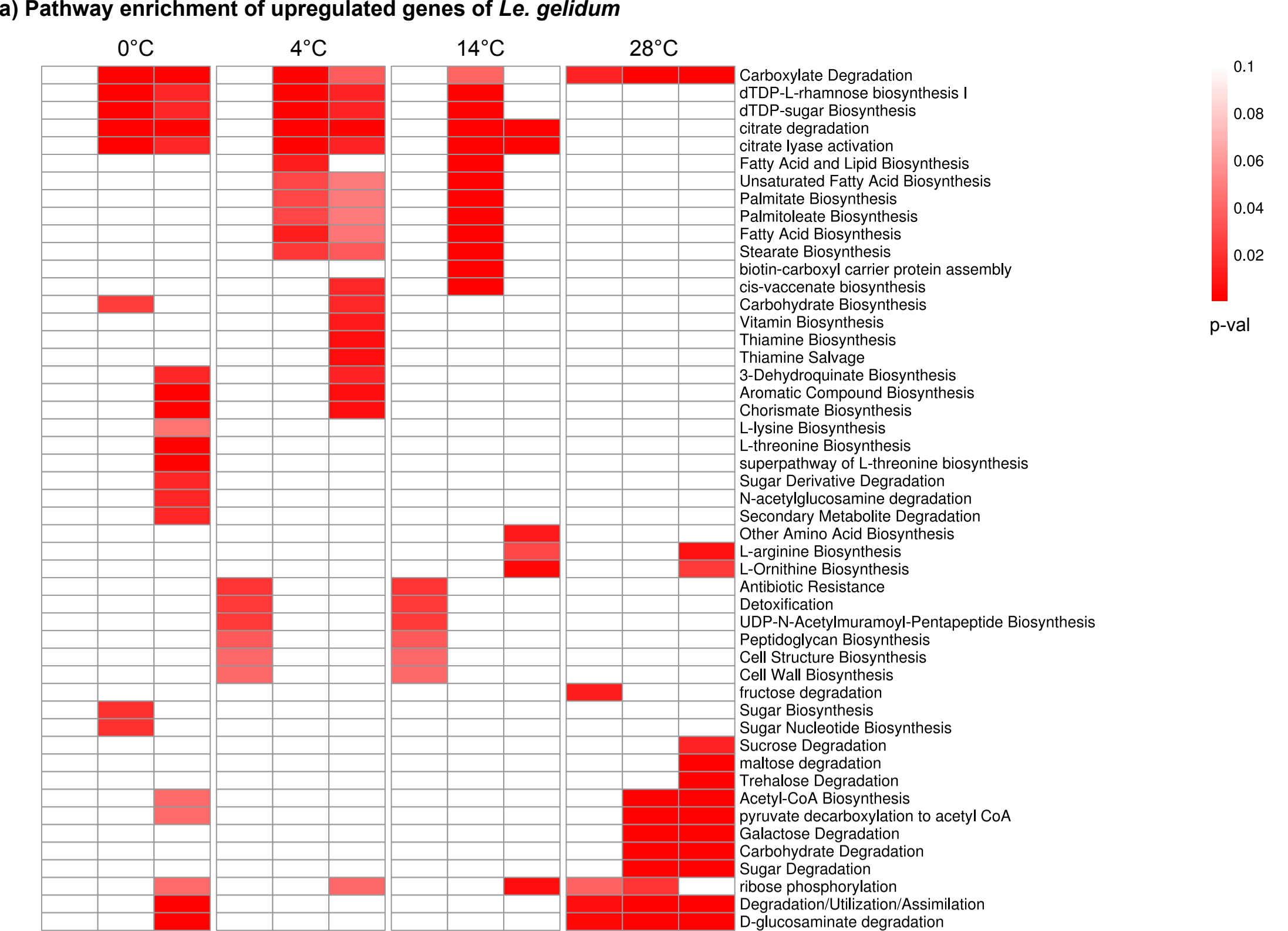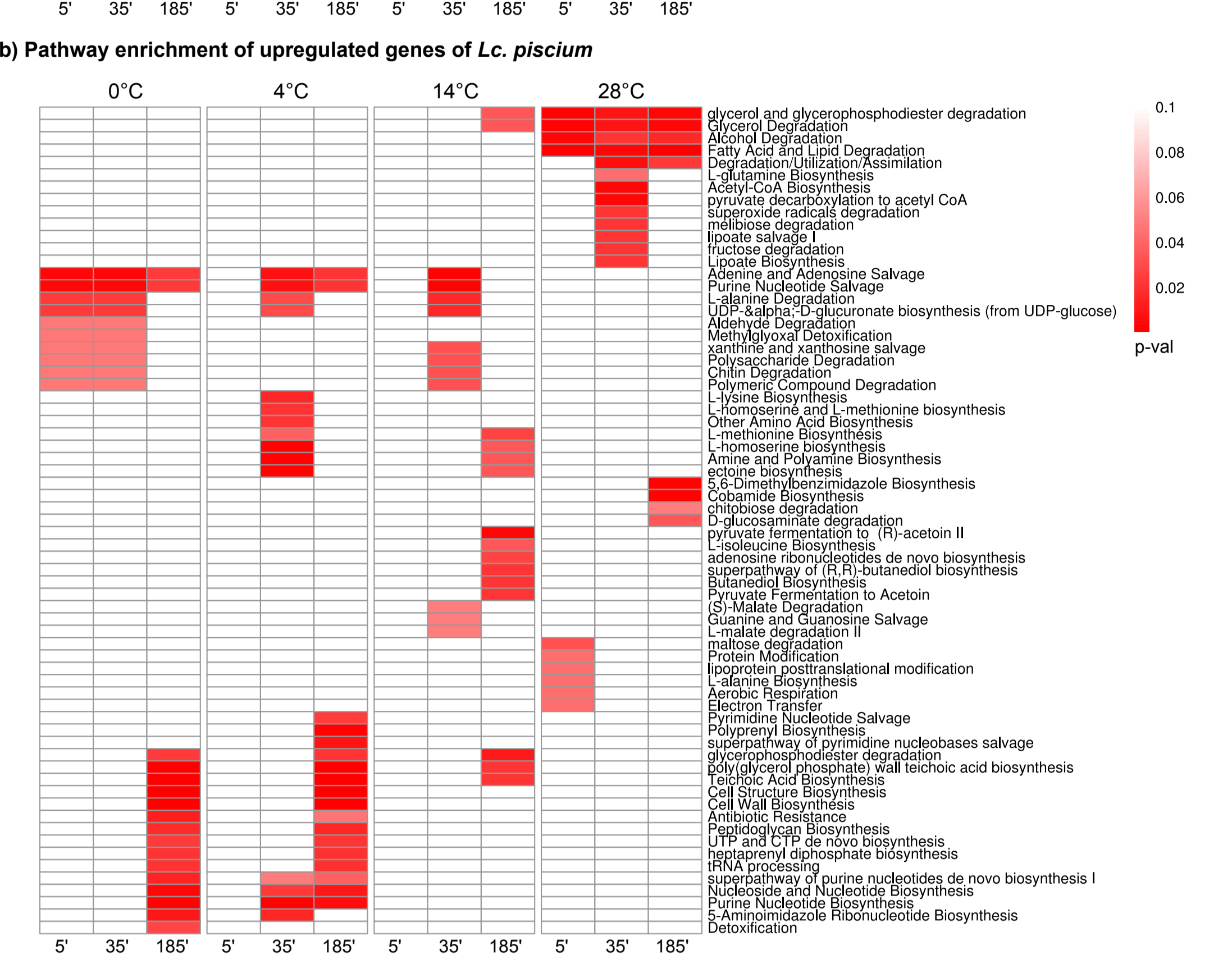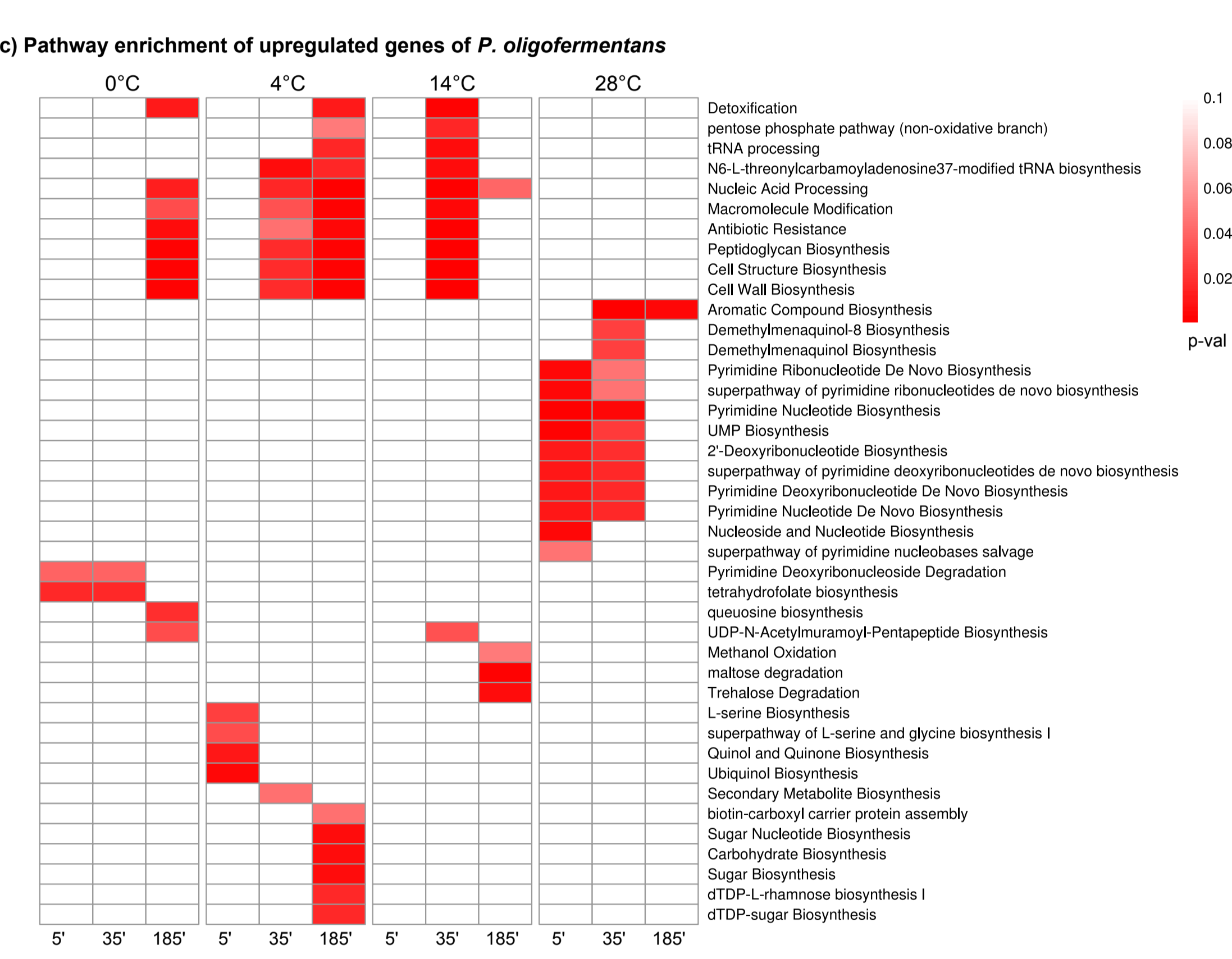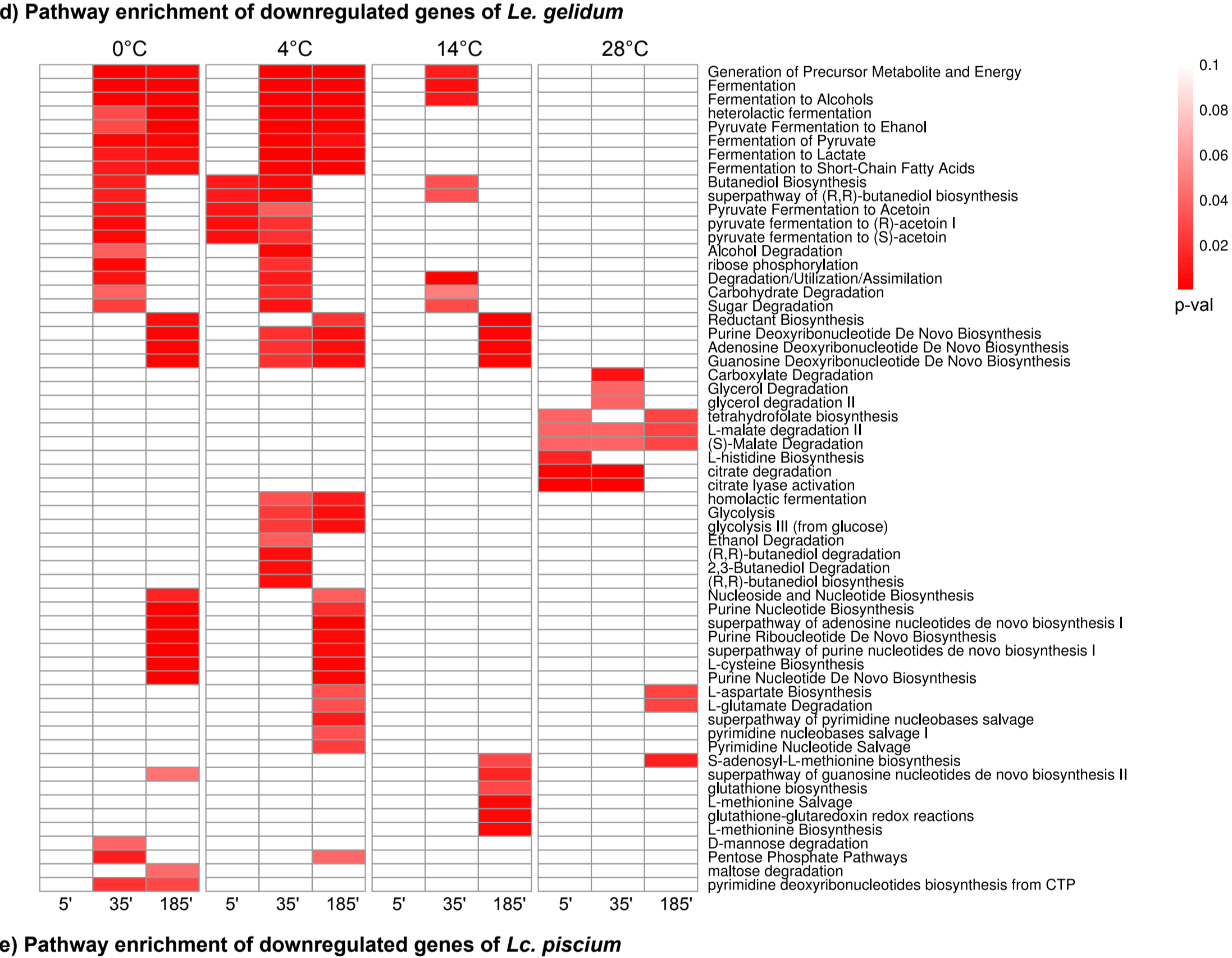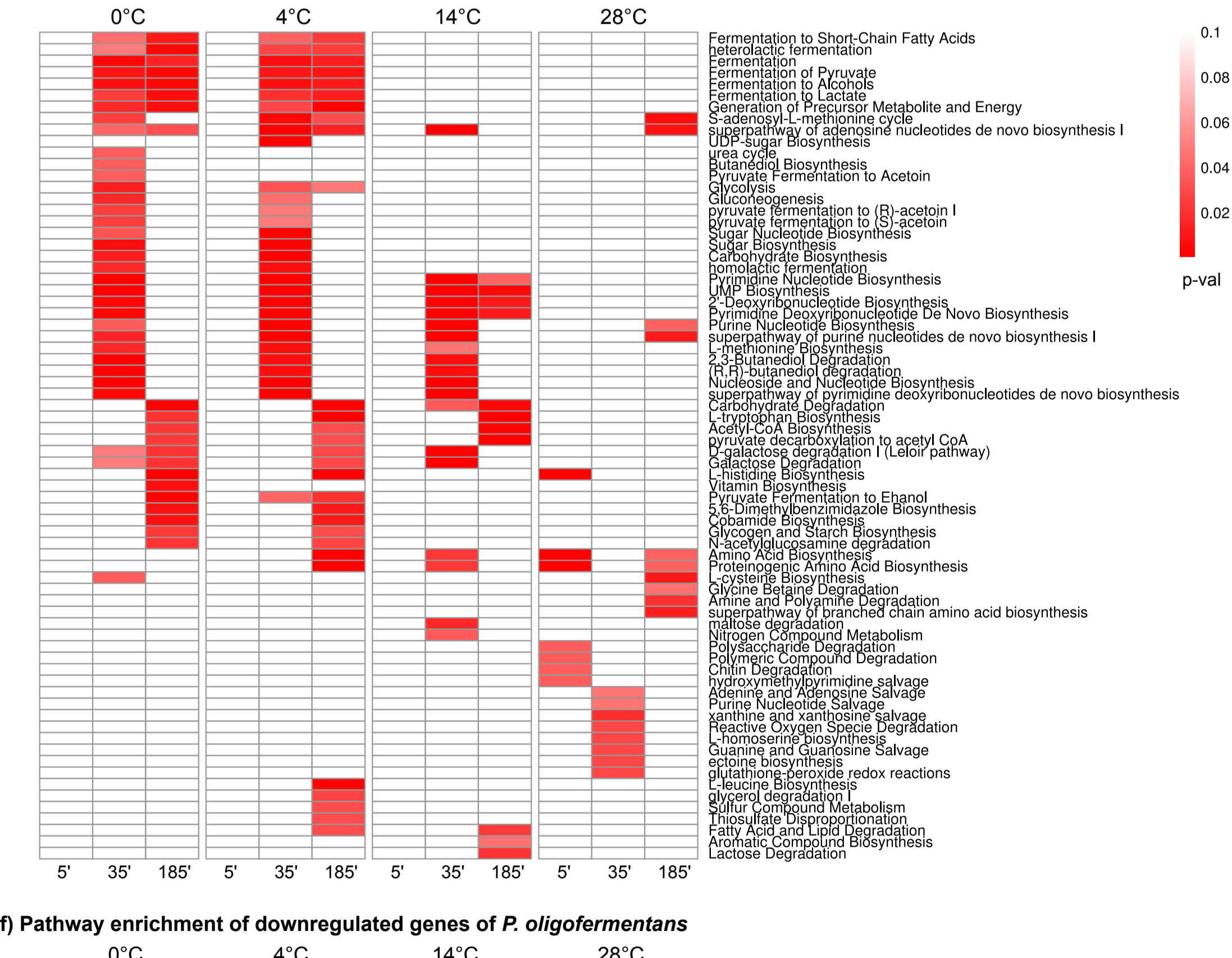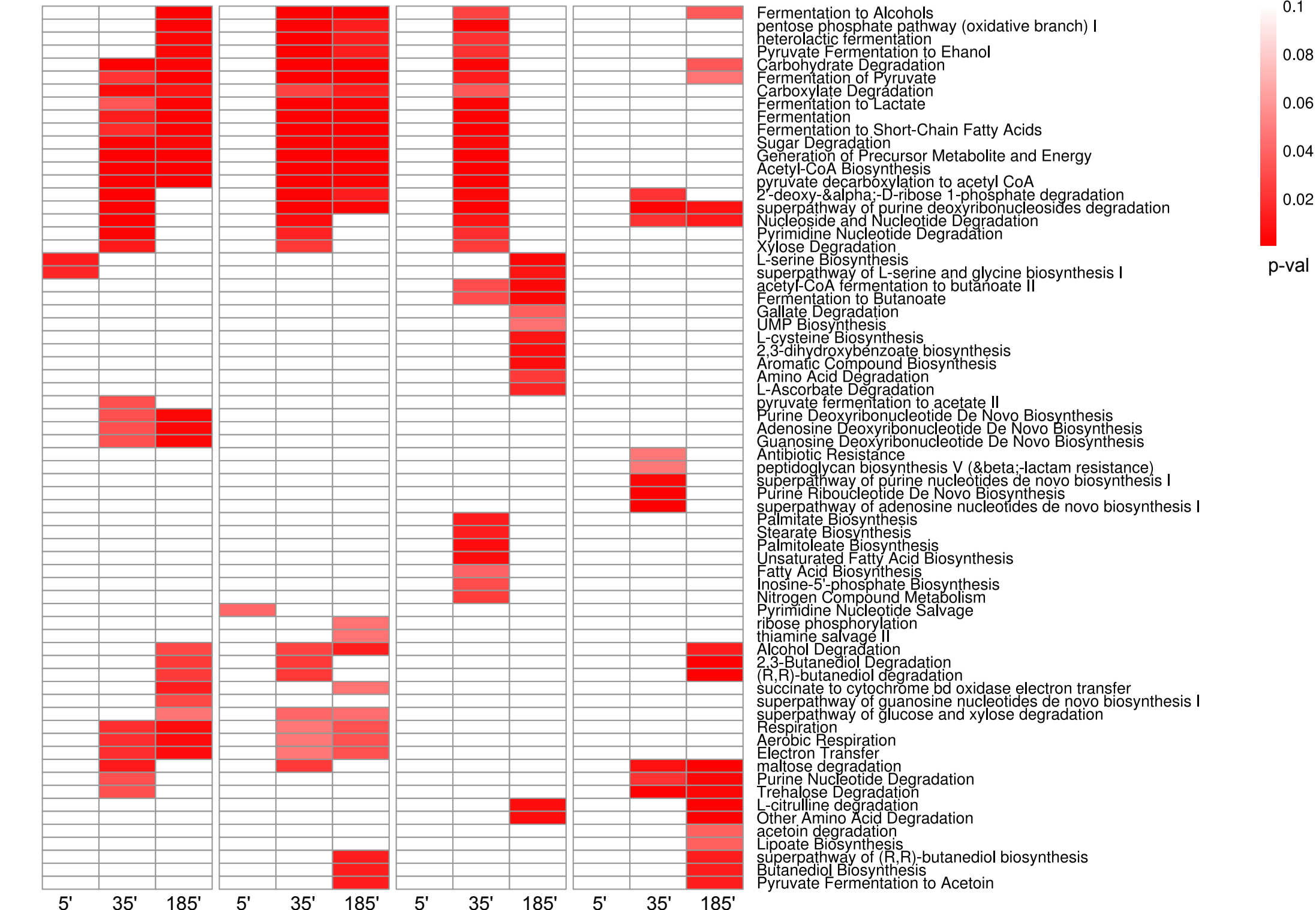

Supplement: Supplementary file 7 — Additional file 7: Figure S3. Metabolic pathway enrichment for upregulated genes of a) Le. gelidum, b) Lc. piscium, c) P. oligofermentans and downregulated genes of d) Le. gelidum, e) Lc. piscium, and f) P. oligofermentans. Figure shows heatmap of enriched metabolic pathways for up- and downregulated genes at different temperatures compared to 25 °C control. Enriched metabolic pathways are marked with red. Red gradient represents enrichment p-value, for which scale is shown at the right corner. The metabolic pathway modelling and metabolic pathway enrichment analysis was performed using Pathway Tools. [file 12864_2020_7338_MOESM7_ESM.pdf]

Pearson's correlation coefficient = 0.94

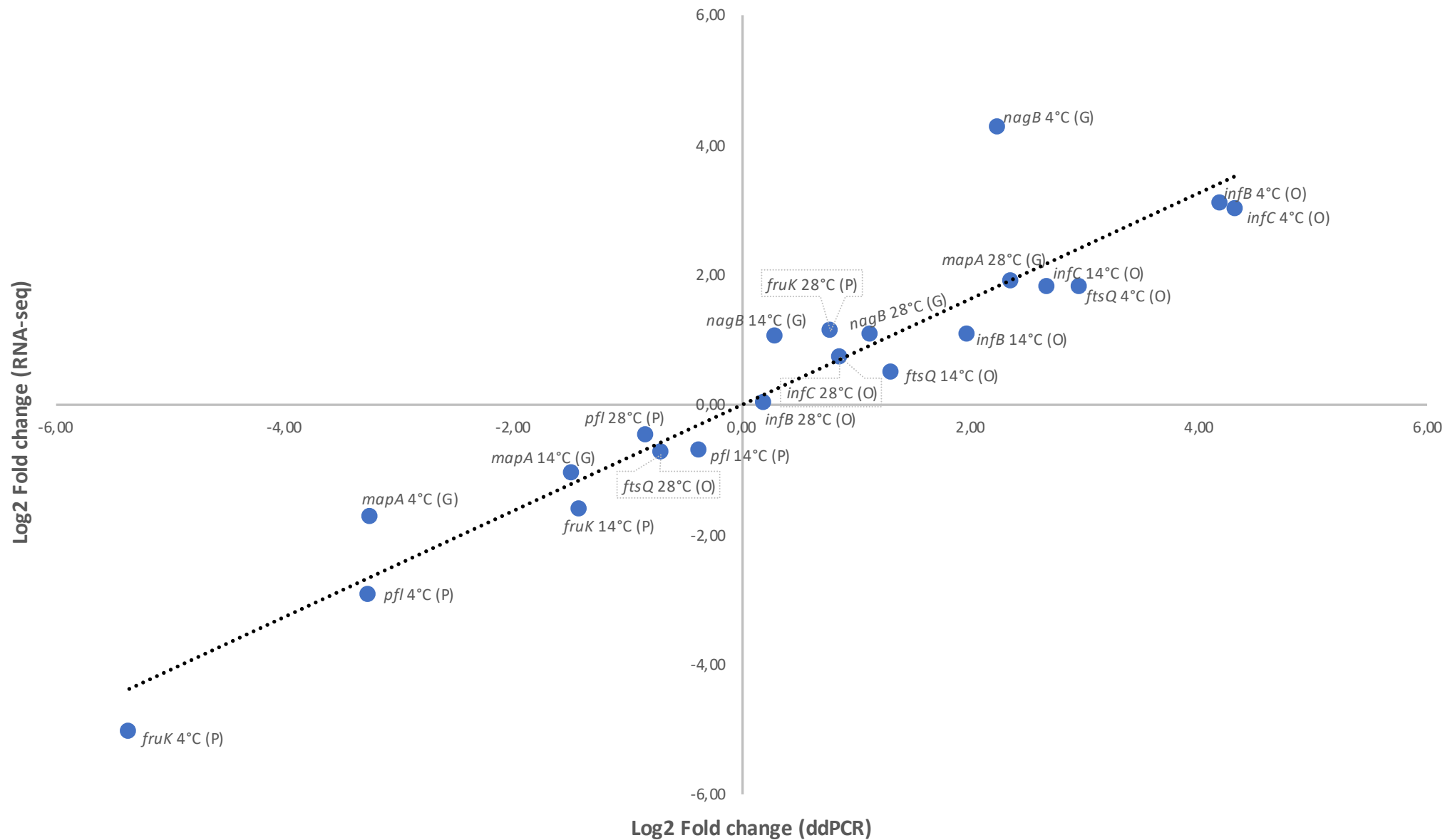

Supplement: Supplementary file 19 — Additional file 19: Figure S5. Comparison of relative expression changes (log2 fold change) for selected genes obtained using ddPCR versus RNA sequencing (RNA-seq). Samples from 185 min were used for the comparison. Temperatures are mentioned after a gene name. A letter in parentheses after a gene name represents the source organism (G, P, and O represents Le. gelidum, Lc. piscium, and P. oligofermentans, respectively). Genes of Le. gelidum; mapA (LEGAS_1151) and nagB (LEGAS_1624) and genes of Lc. piscium; pfl (LACPI_1736) and fruK (LACPI_2020) were normalized using concentration of the housekeeping gene infB. The genes of P. oligofermentans; infC (LACOL_0746), ftsQ (LACOL_1184), infB (LACOL_1061) were normalized using concentration of the 16S rRNA gene. [file 12864_2020_7338_MOESM19_ESM.pdf]

*Le. gelidum*

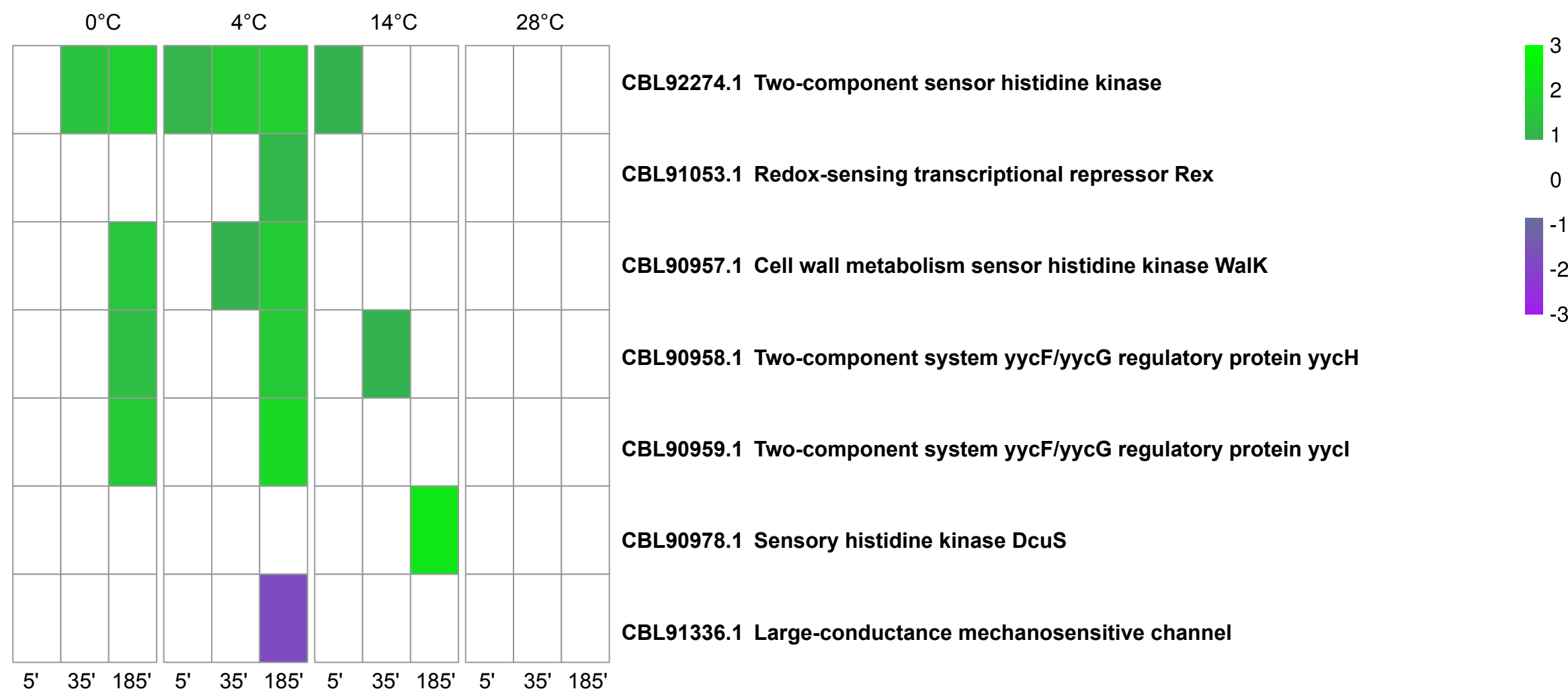

*Lc. pisicum*

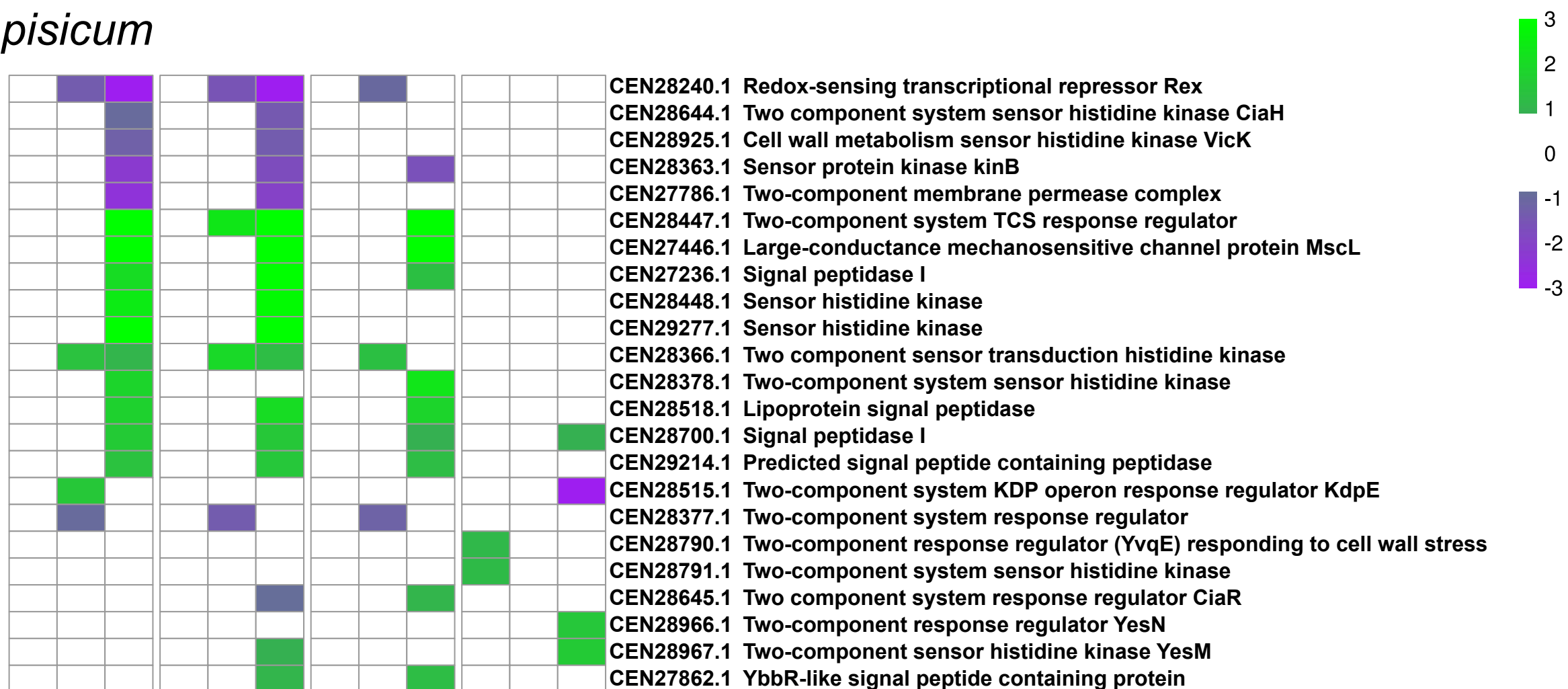

*P. oligofermentans*

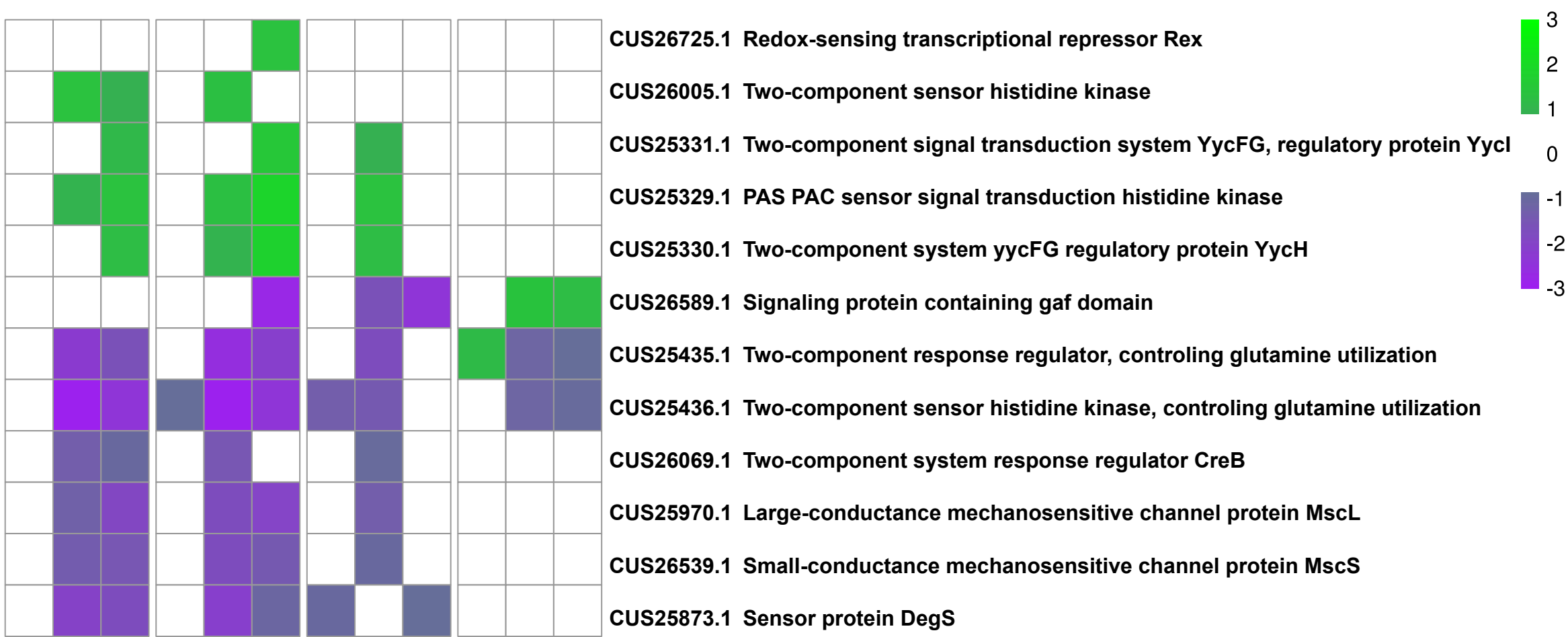

Supplement: Supplementary file 20 — Additional file 20: Figure S6. log2 fold-change heatmap of sensing/signal-related genes in all three species. The log2 fold-change scale is indicated in the right corner. [file 12864_2020_7338_MOESM20_ESM.pdf]
