# Supplementary material for: Transcriptomic time-series analysis of cold- and heat-shock response in psychrotrophic lactic acid bacteria
Source: BMC Genomics. 2021 Jan 7;22:28. doi: 10.1186/s12864-020-07338-8 (PMC7788899; doi:10.1186/s12864-020-07338-8)

a) *Le. gelidum* clustered genes based on gene network inference

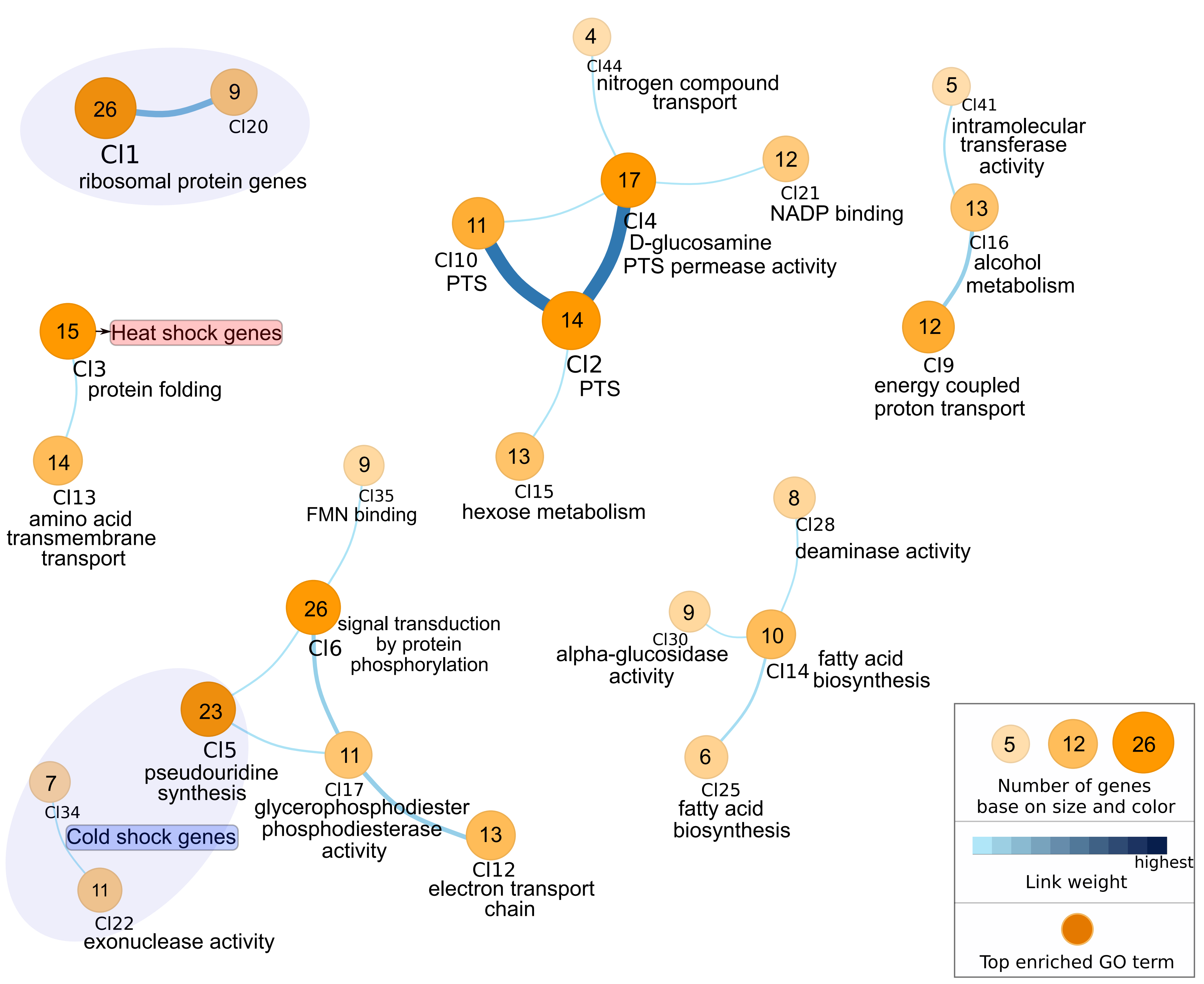

b) *Lc. piscium* clustered genes based on gene network inference

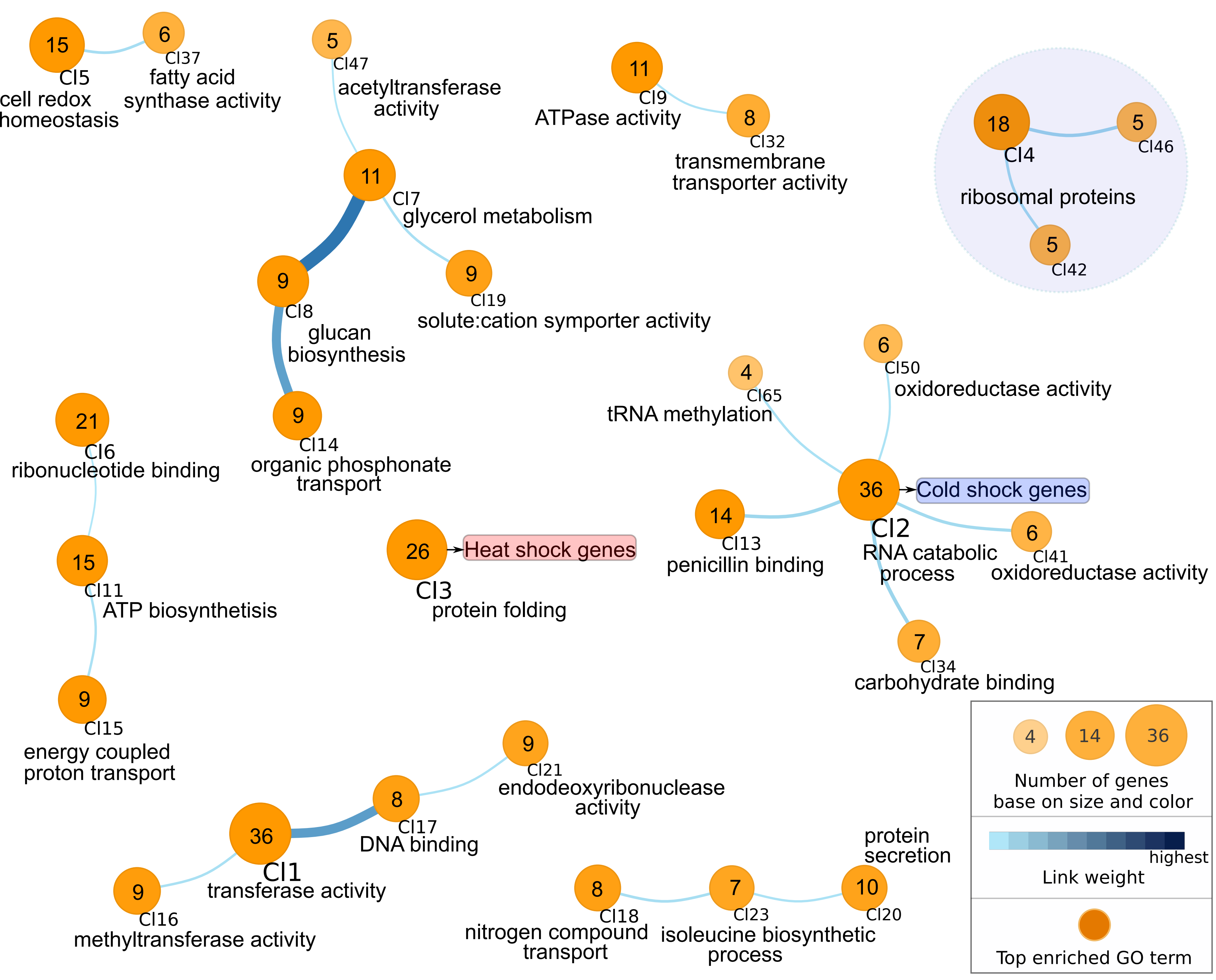

c) *P. oligofermentans* clustered genes based on gene network inference

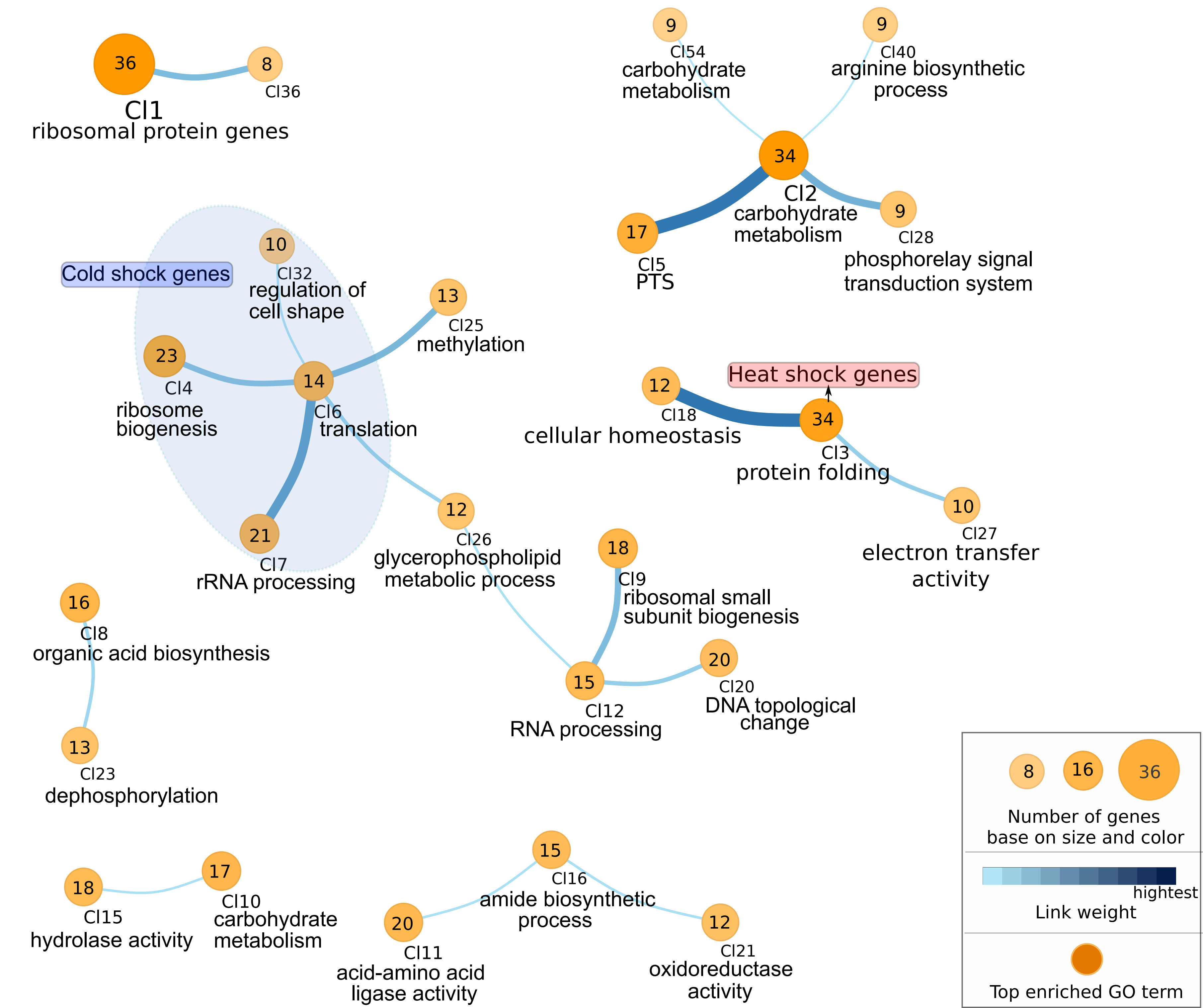

Supplement: Supplementary file 9 — Additional file 9: Figure S4. Clustered genes based on gene network inference in a) Le. gelidum, b) Lc. piscium, and c) P. oligofermentans. Each node represents a cluster and edges represent predicted links between clusters. Number of genes within the cluster is shown in the center of the node. Below the cluster number (Cl), the top-scored enriched GO term is given. Clusters including cold-shock and heat-shock response genes are marked in the figure. Genes within the clusters are listed in Table S5. The scales used are described in the box. For simplification, the figure shows only the top 20 links with the highest weight and the associated connected clusters. [file 12864_2020_7338_MOESM9_ESM.pdf]
